# Design of a Trispecific Checkpoint Inhibitor and Natural Killer Cell Engager Based on a 2 + 1 Common Light Chain Antibody Architecture

**DOI:** 10.3389/fimmu.2021.669496

**Published:** 2021-05-10

**Authors:** Jan P. Bogen, Stefania C. Carrara, David Fiebig, Julius Grzeschik, Björn Hock, Harald Kolmar

**Affiliations:** ^1^ Institute for Organic Chemistry and Biochemistry, Technical University of Darmstadt, Darmstadt, Germany; ^2^ Ferring Darmstadt Laboratory, Biologics Technology and Development, Darmstadt, Germany; ^3^ Global Pharmaceutical Research and Development, Ferring International Center S.A., Saint-Prex, Switzerland

**Keywords:** bispecific antibody, trispecific antibody, NK cell engager, checkpoint inhibitor, common light chain

## Abstract

Natural killer cell engagers gained enormous interest in recent years due to their potent anti-tumor activity and favorable safety profile. Simultaneously, chicken-derived antibodies entered clinical studies paving the way for avian-derived therapeutics. In this study, we describe the affinity maturation of a common light chain (cLC)-based, chicken-derived antibody targeting EGFR, followed by utilization of the same light chain for the isolation of CD16a- and PD-L1-specific monoclonal antibodies. The resulting binders target their respective antigen with single-digit nanomolar affinity while blocking the ligand binding of all three respective receptors. Following library-based humanization, bispecific and trispecific variants in a standard 1 + 1 or a 2 + 1 common light chain format were generated, simultaneously targeting EGFR, CD16a, and PD-L1. The trispecific antibody mediated an elevated antibody-dependent cellular cytotoxicity (ADCC) in comparison to the EGFR×CD16a bispecific variant by effectively bridging EGFR/PD-L1 double-positive cancer cells with CD16a-positive effector cells. These findings represent, to our knowledge, the first detailed report on the generation of a trispecific 2 + 1 antibodies exhibiting a common light chain and illustrate synergistic effects of trispecific antigen binding. Overall, this generic procedure paves the way for the engineering of tri- and oligospecific therapeutic antibodies derived from avian immunizations.

## Introduction

With the FDA approval of blinatumomab in 2014, bispecific antibodies (bsAb), particularly those bringing immune cells in spatial proximity to malignant tumor cells, raised further interest for therapeutic application (1, 2). Their unique mechanism is based on the simultaneous binding of a cancer-specific antigen on the surface of a tumor cell and a specific marker on the surface of immune cells, activating the latter, leading to efficient killing of the malignant cells. Blinatumomab is a tandem single-chain fragment variable (scFv) targeting CD19 on B cells of acute lymphoblastic leukemia (ALL) patients and links them *via* its CD3 binding moiety to cytotoxic T cells (3). Even though blinatumomab was granted breakthrough therapy for the treatment of ALL, its therapeutic usage is limited by the short half-life of the molecule, leading to the need for continuous infusion (4). Furthermore, the high potency of bispecific T-cell engager (BiTE) molecules is associated with increased toxicity, resulting in a narrow therapeutic window (5–9).

To overcome the toxic effects of T cell engagers, the concept of natural killer (NK) cell engagers was created, based on their anti-tumor activity (10, 11). NK cells express CD16a, also known as FcγRIIIa, which binds with low affinity to the Fc parts of antibodies (12, 13). Furthermore, engagement of CD16a is less demanding compared to CD3 engagement due to lower steric hindrances and additionally facilitated by the lack of accessory molecules (14).

Upon recognizing a target cell decorated with antibodies, NK cells mediate antibody-dependent cellular cytotoxicity (ADCC) resulting in killing of target cells (15, 16). This naturally occurring mechanism was utilized by Wiernik and coworkers to generate a CD16×CD33 bispecific killer cell engager (BiKE) showing effective engagement of CD33-positive cells by NK cells, resulting in cytotoxic effects (17). This concept was further optimized by implementing an IL-15 moiety within the linker between both scFvs, resulting in a trispecific killer cell engager (TriKE). The additional IL-15 moiety mediated superior NK cell cytotoxicity, degranulation and resulted in increased NK cell proliferation and survival (18).

Recently, Gauthier et al. introduced trifunctional natural killer cell engagers (NKCEs) co-engaging not only CD16 but also NKp46, another activating NK cell receptor, and a tumor-specific antigen, yielding impressive outcomes in *in vitro* and *in vivo* experiments while exhibiting an improved safety profile when compared to BiTEs (19).

The epidermal growth factor receptor (EGFR), a member of the ErbB family, is expressed in a variety of cancers, including lung cancer, bladder cancer, and colorectal cancer, where it is associated with tumor progression and metastasis (20). Upon binding to its receptor, the epidermal growth factor (EGF) promotes cell proliferation and survival (21). Multiple bispecific diabodies targeting EGFR×CD16a have been engineered (14, 22) and recently, AFM24, a tetravalent bispecific EGFR×CD16a targeting molecule, entered clinical testing in a phase I/II study (NCT04259450). Even though BiKEs and TriKEs exhibit extraordinary favorable properties, their therapeutic usage is limited by their short half-life.

Notably, NK cell activity can be negatively influenced by immune checkpoints (23). The PD-1/PD-L1 axis is of major interest as it is an immune checkpoint for T cells (24–26) as well as for NK cells (27–29). Originally described as an immune checkpoint for T lymphocytes, the inhibition of the PD-1/PD-L1 axis showed tremendous effects in clinical applications (30–32). In many malignant cancers, PD-L1 is upregulated to overcome the immune surveillance (33, 34). EGFR, on the other hand, is naturally expressed on epithelial cells in the skin and the lung (35–37), but becomes overexpressed in many tumors of epithelial origin, where it mediates cell proliferation and survival. This lack of tumor specificity accounts for on-target/off-tumor side effects in immunotherapeutic treatments (38–40). Koopmans and coworkers generated a bispecific EGFR×PD-L1 antibody, blocking the PD-L1 immune checkpoint in an EGFR-dependent manner, resulting in a potentially favorable safety profile. Bispecific EGFR×PD-L1 antibodies showed a superior tumor uptake compared to the MOCK×PD-L1 control antibody in xenografts (41).

As EGF signaling can induce PD-L1 upregulation in tumor cells, thereby shielding the tumor from the immune system, co-expression of both proteins occurs predominantly on cancer cells (42–44). To specifically target those cells, we strived to generate a trispecific anti-EGFR×CD16a×PD-L1 antibody. We chose an Fc-based approach, due to the significantly prolonged half-life mediated by the size, which prevents renal clearance, and the recycling process mediated by the binding to the neonatal Fc receptor (FcRn).

Bacac and coworkers generated a trivalent, bispecific CEA×CD3×CEA antibody (45). In this 2 + 1 molecule, an additional anti-CEA Fab was N-terminally fused to the anti-CD3 Fab of a bispecific 1 + 1 CEA×CD3 antibody. Heterodimerization of the heavy chains was ensured by the Knob-into-Hole (KiH) technology, while light chain pairing was mediated by the CrossMab technology (46, 47). The resulting 2 + 1 T-cell bispecific (TCB) antibody was termed cibisatamab and is currently in phase I clinical testing (NCT03866239).

**Figure 1:**
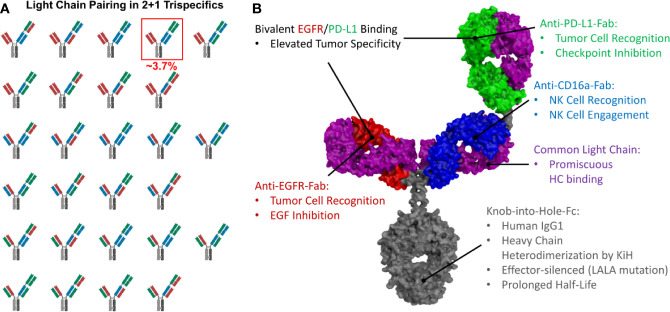
Possible light chain pairing combinations and structural model of trispecific antibodies. **(A)** In 2 + 1 trispecifics, 27 different light chain pairing combinations are possible, resulting in only ~3.7% correctly paired antibodies. The correctly paired variant is highlighted. **(B)** Structural model of a 2 + 1 trispecific antibody exhibiting common light chains to circumvent light chain mispairing. Intended functionalities are depicted.

Based on that design, we aimed to generate a trispecific antibody based on a bispecific EGFR×CD16a antibody. The anti-PD-L1 arm should be N-terminally fused to the CD16a-binding Fab (**Figure 1**), as the immune engaging moiety displays a favorable safety profile in this “inner” position (48–50). While the EGFR-specific Fab blocks EGF signaling, the PD-L1-specific Fab can inhibit immune checkpoints and, in combination, both could facilitate an enhanced tumor selectivity. Binding to EGFR/PD-L1 may mediate clustering on target cells, leading to efficient CD16a clustering on effector cells and, therefore, results in potent cytotoxic activity.

The heterodimerization of the heavy chains can easily be achieved by utilizing the Knob-into-Hole technology (51). However, light chain pairing remains a major challenge. While bispecific 1 + 1 antibodies yield 25% of correctly paired antibodies if the light chain association is non-directed (**Supplementary Figure 1**), in trispecific 2 + 1 antibodies, only ~3.7% of the resulting antibodies are correctly paired (**Figure 1A**). Technologies like orthogonal Fab interfaces (52) or the CrossMab technology (46) can be used to circumvent this issue, but the most straightforward approach is the utilization of a common light chain, which pairs with all three VH-CH1 moieties and still results in fully functional binding entities (**Figure 1B**).

We recently showed that immunization of chickens enables the isolation of common light chain antibodies targeting a broad epitope space (53), and that these antibodies can be assembled in a heterodimerized manner resulting in biparatopic antibodies comprising cLCs (54). Furthermore, we demonstrated a straightforward method for humanization of chicken-derived molecules (55). Here, we describe the affinity maturation of an anti-EGFR cLC-based antibody by light chain shuffling and utilization of the resulting light chain as a new common light chain to generate anti-CD16a and anti-PD-L1 antibodies. After humanization, we generated the first humanized bi- and trispecific chicken-derived antibodies, simultaneously targeting EGFR, CD16a, and PD-L1 resulting in a strong antibody-dependent cytotoxic effect.

## Results

### Anti-EGFR Fab: Affinity Maturation by Light Chain Shuffling

Recently, we isolated a chicken-derived EGFR-specific antibody termed FEB4 from an immune library comprising a restricted light chain diversity. The respective antibody showed affinity in the lower-double-digit nanomolar range, targeted a conformational epitope on EGFR domain III, exhibited an overlapping binding site with matuzumab, and inhibited EGF binding to its receptor (54). Due to its ability to block EGF binding to its receptor, as well as targeting a proximal binding site to the intensively investigated matuzumab epitope, we chose FEB4 as a starting molecule.

Since yeast surface-displayed (YSD) FEB4 showed significant binding to EGFR even at 1 nM antigen concentration when paired with other unrelated light chains (54), we thought of a light chain shuffling-based affinity maturation approach.

**Figure 2:**
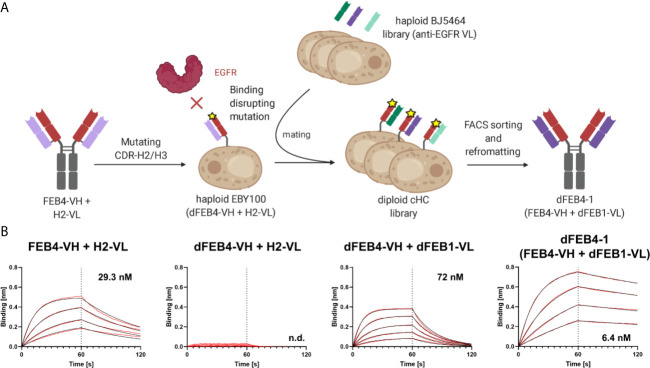
Affinity maturation of EGFR-binding FEB4 **(A)** Schematic representation of light chain shuffling approach. The mutations in the CDR-H2 and CDR-H3 disrupt the binding to EGFR. By shuffling with a light chain immune repertoire derived from EGFR-immunized chicken by yeast mating and FACS sorting, an affinity maturated variant was isolated. **(B)** BLI affinity measurements of FEB4-derived antibodies at different maturation steps.

In this approach, the antigen binding of the heavy chain is strongly reduced by introducing mutations to the CDRs. Subsequently, by pairing this impaired heavy chain with a diverse light chain library, variants with restored binding ability might be isolated, where light chain residues compensate for the disrupted heavy chain CDRs. If this light chain is subsequently paired with the original heavy chain, the additional binding-mediating residues of the light chain could lead to higher affine binding (**Figure 2A**).

To test this hypothesis, two residues in the CDR-H2 (D54E and T58V) and two residues in the CDR-H3 (N105Q and D109E) were substituted, giving rise to the disrupted-FEB4-VH (dFEB4), resulting in nearly eradicated antigen binding (**Supplementary Figure 2A**). Nonetheless, antibody production yields as a full-length IgG molecule, as well as its ability to display on yeast cells, remained unchanged after residue replacement. The mutations were chosen to have no major impact on electrostatic charge or hydrophobicity of the paratope, but only introduce steric hindrances to prevent EGFR binding.

In order to generate a light chain library, the VL genes were amplified from cDNA derived from the EGFR-immunized chicken from which the FEB4 VH gene originated (54, 56). The genes for VL domains were inserted into a pYD1-derived vector encoding a human lambda CL by homologous recombination in BJ5464 yeast. Library generation resulted in 2.9×10^8^ transformants, ensuring sufficient oversampling of the potential diversity. Subsequently, this light chain diversity in BJ5464 yeast cells was combined with EBY100 yeast cells encoding the dFEB4 VH-CH1 transcript by yeast mating. The resulting diploid common heavy chain library was screened for binding to EGFR-ECD-Fc chimera *via* FACS (**Supplementary Figure 2B**).

Following the first sorting round, a large population of yeast cells displaying EGFR-specific antibodies was enriched. A kinetic off-rate screening utilizing 1 nM EGFR-ECD-Fc competing with 1 µM His-tagged EGFR-ECD was performed to ensure the isolation of affine variants. Five unique antibody variants were identified and analyzed by flow cytometry for EGFR binding (**Supplementary Figure 3A**). All variants showed a significant binding at low antigen concentrations and exhibited diverse VL sequences comprising different CDR lengths and amino acid compositions (**Supplementary Figure 3B**). VL sequences were reformatted into pTT5-derived vectors for mammalian cell expression encoding the lambda CL sequence by Golden Gate Cloning and co-transfected in Expi293F cells with a pTT5 vector encoding the parental FEB4 heavy chain. Biolayer interferometric (BLI) measurements of sterile filtered cell culture supernatants revealed that, except for one variant, all light chains mediated an elevated affinity compared to the parental antibody (**Supplementary Figures 3C, D**). The variant with the highest affinity was chosen, which exhibited an affinity of 6.4 nM after Protein A chromatography purification, representing a five-fold improvement in KD value (**Figure 2B**). This heavy chain – light chain combination was termed dFEB4-1.

Subsequently, dFEB4-1 was analyzed *via* size exclusion chromatography (SEC), resulting in an improved profile compared to the parental variant since no aggregates could be detected (**Supplementary Figure 3E**). Thermal stability was analyzed using NanoDSF, showing that dFEB4-1 exhibited a slightly higher T_M_ value compared to the parental antibody (**Supplementary Figure 3F**).

**Figure 3:**
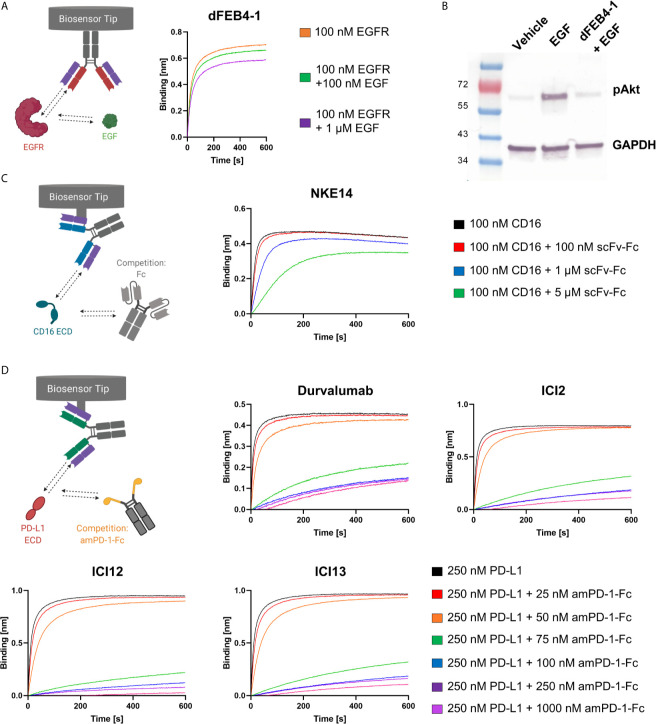
Ligand-receptor blockage of isolated cLC-mAbs. **(A)** BLI-assisted EGF-inhibition assay. Immobilized dFEB4-1 binds to EGFR at different EGF concentrations, revealing a dose-dependent binding. **(B)** Western blot analysis of inhibition of EGF-induced phosphorylation of AKT in A549 cells. 50 µg/mL dFEB1-4 and 10 ng/mL EGF were utilized. The western blot was performed three times, yielding reproducible results. **(C)** Epitope determination of NKE14. Immobilized NKE14 binds to CD16a at different scFv-Fc concentrations. CD16a:Fc-binding led to diminished binding of NKE14 to CD16a in a dose-dependent manner. **(D)** PD-1:PD-L1 interaction inhibition assay. Immobilized ICI2, ICI12, ICI13, or durvalumab binding to PD-L1 at different amPD-1-Fc concentrations, revealing dose-dependent binding.

Biolayer interferometric measurements revealed no epitope drift, as dFEB4-1 targeted the same epitope as the parental FEB4 mAb, exhibiting an overlapping epitope with matuzumab, but not with cetuximab (**Supplementary Figure 4**). These results are in accordance with previous binning experiments performed with the parental antibody variant (54). Additionally, BLI measurements showed that EGF interferes with the ability of dFEB4-1 to bind EGFR (**Figure 3A**), comparable to the parental molecule or cetuximab (54). Furthermore, dFEB4-1 was able to inhibit EGF-induced phosphorylation of AKT in EGFR-positive A549 cells (**Figure 3B**).

Taken together, these results indicate that dFEB1-VL exhibits more favorable biophysical properties in terms of affinity and stability, while no epitope drift was observed. Therefore, dFEB1-VL was chosen as a novel common light chain for all subsequent sorting approaches to isolate binding molecules against CD16a and PD-L1.

### Chicken Immunization and Library Generation

CD16a for chicken immunization was produced in-house as two variants. The first one was a bivalent N-terminal Fc-fusion, where the Fc exhibited the P329G LALA and the N297A mutation to circumvent Fc-binding by the CD16a moiety, which would have led to aggregation (57, 58). The second variant was a monomeric CD16a protein with an N-terminal His-tag and a C-terminal TwinStrep-tag. Both constructs were expressed in Expi293F cells and purified either using Protein A chromatography or Strep-Tactin XT columns, respectively according to the manufacturer’s description. Immunization was started utilizing Fc-tagged CD16a, and after two doses, the animal was boosted with monomeric CD16a, resulting in high titer values for both antigens. Additionally, a second chicken was immunized with commercially available PD-L1-Fc chimera (PeproTech), also leading to a high antibody titer after four immunizations (**Supplementary Figure 5**).

Library generation yielded in 7.1×10^9^ or 6.5×10^9^ transformed yeast cells for the CD16a and PD-L1 libraries, respectively. Simultaneously, the dFEB1-VL light chain-encoding pYD1 plasmid was transformed into BJ5464 yeast cells. Both haploid yeast cell populations were subsequently mated, resulting in diploid yeast cells displaying VH domains from either the CD16a- or the PD-L1-immunized chicken, paired with the previously described dFEB1-VL common light chain.

### Anti-CD16 Fab Library: Screening and Characterization

The yeast library was sorted over three rounds against CD16a-Fc and monomeric CD16a, resulting in the enrichment of a binding population (**Supplementary Figure 6A**). Sequence analysis of twenty randomly chosen clones revealed 17 different VH sequences. All variants were reformatted using Golden Gate Cloning as described before (53), utilizing an aglycosylated N297A Fc to circumvent CD16a:Fc-interactions. All 17 variants were loaded onto AHC BLI biosensors and tested for specific antigen binding. The best-performing variant NKE14 specifically bound both human CD16a isoforms with 5.6 nM or 5.9 nM affinity, respectively (**Supplementary Figure 6B**). Furthermore, NKE14 exhibited an excellent aggregation profile in SEC experiments, as well as a notable T_M_ value as determined by NanoDSF (**Supplementary Figures 6C, D**).

*In vivo*, CD16a recognizes Fc portions of target-bound IgG1 antibodies, leading, upon CD16a clustering, to an efficient ADCC response. To investigate, whether the epitope of NKE14 on CD16a overlaps with the Fc recognition site, an epitope binning experiment in the premixed setup was performed in a dose-dependent manner. NKE14 was loaded onto Fab2G biosensors and was subsequently exposed to high binding CD16a 176V isotype pre-incubated with different molar ratios of an unrelated scFv-Fc fusion, which contained a glycosylated Fc, allowing for CD16a binding. The binding of the Fc fusion by CD16a impaired the binding of NKE14 to CD16a in a dose-dependent manner, indicating that NKE14 binds to an epitope that is also involved in the interaction of CD16a with Fc regions (**Figure 3C**).

### Anti-PD-L1 Fab Library: Screening and Characterization

The PD-L1 library was sorted for two rounds utilizing consecutively 1 µM and 250 nM of His-tagged PD-L1 (R&D systems) until a clear enrichment of binders was observed (**Supplementary Figure 7A**). In order to isolate mAbs capable of inhibiting the interaction between PD-L1 and PD-1, an epitope binning-based screening was applied as previously described (54). Therefore, the enriched library was stained with 10 nM biotinylated PD-L1 to ensure the staining of high affine binders. To isolate only antibodies able to block the interaction between PD-L1 and PD-1, 100 nM of the FDA-approved anti-PD-L1 antibody durvalumab with a known epitope was applied to the library. Double-positive yeast cells for PD-L1 and durvalumab are indicative for surface-displayed Fabs that target a non-overlapping epitope with durvalumab (**Supplementary Figure 7B**). Conversely, the sorting gate was applied to isolate yeast cells exhibiting a chicken-derived Fab fragment that does bind PD-L1 but is not interacting with durvalumab, as those are expected to compete with durvalumab for binding to related epitopes on PD-L1 (**Supplementary Figures 7C, D**). VH genes of this population were amplified after plasmid isolation and reformatted into IgG1 LALA chimeric antibodies by Golden Gate cloning. Sanger sequencing revealed five unique clones that were subsequently produced in Expi293F cells and purified by Protein A chromatography. Three out of these five immune checkpoint inhibitor variants (ICI), termed ICI2, ICI12 and ICI13, bound PD-L1 with affinities ranging from 3.0 nM to 5.8 nM, and therefore the non-binding variants ICI7 and ICI15 were omitted from further analysis (**Supplementary Figure 8A**). Biophysical characterization showed excellent aggregation behavior of all three remaining variants, as well as prominent thermal stabilities (**Supplementary Figures 8B, C**).

For further functional characterization, an affinity-matured PD-1 variant (amPD-1/HAC-V), which was described by Maute and coworkers (59), was produced as a bivalent Fc-fusion protein in Expi293F cells and purified by Protein A chromatography. BLI experiments revealed an affinity of 3.5 nM towards PD-L1, underlining its significantly elevated affinity compared to wild type PD-1, with a KD value determined to be between 4 µM and 7 µM (**Supplementary Figure 8A**) (60, 61). This high affine variant was utilized to measure antibody-mediated ligand receptor blockage.

Fab2G tips were loaded with the respective anti-PD-L1 antibodies, either chicken-derived or durvalumab as a positive control, and subsequently associated to 250 nM PD-L1 preincubated with different concentrations (0 nM up to 1000 nM) of amPD-1-Fc. All isolated antibodies showed significantly impaired binding to PD-L1 in the presence of amPD-1-Fc in a dose-dependent manner comparable with durvalumab, indicating that all isolated antibodies target the interaction site of PD-1 and PD-L1 (**Figure 3D**).

Furthermore, an epitope binning in the in-tandem setup was performed with PD-L1 immobilized on Ni-NTA biosensors, followed by ICI2, ICI12, ICI13, or durvalumab. Subsequently, all mAbs of interest and amPD-1-Fc were applied, revealing that all antibodies share an overlapping epitope with each other and the PD-1 interaction site, confirming the YSD-assisted epitope binning-based screening as well as the blockage assay (**Supplementary Figure 9**). Since ICI2 revealed the highest affinity towards PD-L1 and exhibited promising blocking properties, it was chosen for further engineering.

### Humanization

For therapeutic usage, avian-derived antibodies are unsuitable due to their high immunogenicity, likely resulting in anti-drug-antibodies (ADA), which diminish the effectiveness of therapeutic mAbs (62). To circumvent this problem, our group previously established a YSD-based humanization strategy (55). In brief, the CDRs of the chicken-derived antibody are grafted onto a human acceptor framework with simultaneous randomization of Vernier residues, critical for CDR orientation. A YSD library is generated and displayed on the surface of yeast cells. The most affine humanized variants can be isolated *via* FACS.

Since dFEB4-1, NKE14, and ICI2 all comprise an identical common light chain, a simultaneous humanization might not lead to identical light chains regarding the Vernier residues. Therefore, dFEB4-1 was humanized first. Over two rounds of sorting, including a kinetic off-rate screening, humanized variants were enriched (**Supplementary Figure 10A**). Of ten randomly chosen clones, seven unique variants were identified, which were subsequently reformatted into a chimeric IgG1 format and verified for effective target binding (**Supplementary Figure 10B**). The variant hdFEB4-1-4 displayed the highest affinity with 23.5 nM. It was analyzed in a SEC experiment and revealed an excellent aggregation profile (**Supplementary Figure 10C**). Additional measurements of thermal stability underlined the notable stability. (**Supplementary Figure 10D**). The light chain of hdFEB4-1-4 was therefore chosen as a fixed common light chain for the further humanization of the VH domains of NKE14 and ICI2. Therefore, these libraries only comprised randomized Vernier residues in the VH domains.

For both libraries, three rounds of sorting with decreasing concentration of the respective antigen were sufficient to enrich humanized binders (**Supplementary Figures 11A, B**). Plasmids were isolated from the enriched populations, VH sequences were amplified by PCR and subsequently inserted into pTT5-derived expression vectors exhibiting an IgG1 LALA Fc by Golden Gate cloning. Sequencing revealed three unique humanized variants for the hNKE14-library and six unique variants for the hICI2-library. All respective variants were produced in Expi293F cells and purified by Protein A chromatography, followed by subsequent affinity determination using BLI (**Supplementary Figures 12A, B**). The hNKE14 variants displayed affinities in the range of 15.5 nM to 25.7 nM, while the hICI2-variants exhibited binding affinities between 1.8 nM and 3.2 nM, which was marginally elevated compared to the parental ICI2 chicken-derived mAb. Based on their KD values, as well as excellent aggregation behavior and high thermal stability (**Supplementary Figures 12C–F**), hNKE14-8 and hICI2-3 were chosen for subsequent use.

### Construction and Characterization of Bispecific and Trispecific cLC Antibodies

The humanized Fab fragments were subcloned into a bispecific 1 + 1 and a trispecific 2 + 1 format *via* Golden Gate Cloning. The Fc fragments exhibited a Knob-into-Hole mutation to force heterodimerization of the heavy chains. Furthermore, a His-tag was cloned C-terminally to the Hole- and a TwinStrep-tag was C-terminally fused to the Knob-Fc to allow a two-step purification yielding only correctly assembled heterodimeric antibodies as previously described (54). As controls, one-armed hdFEB4-1-4 and hNKE14-8 variants were cloned following identical architecture. To circumvent CD16a interaction with the Fc, the LALA mutation was applied to all produced heterodimeric antibodies. All variants were produced in Expi293F cells and purified by IMAC, followed by StrepTactin XT chromatography. Furthermore, monovalent one-armed (oa) variants of hdFEB4-1-4 (oahdFEB4-1-4) and hNKE14-8 (oahNKE14-8), exhibiting His- and TwinStrep-tags, were cloned and produced identically.

SDS-PAGE analysis under reducing conditions showed the expression of similar amounts of both heavy chains, indicating correct pairing of all produced bi- and trispecific molecules (**Supplementary Figure 13A**). Additionally, a SDS-PAGE under non-reducing conditions was performed, revealing a single band at ~200 kDa, which is 50 kDa, the size of a single Fab arm, larger compared to the bispecific mAb under the same conditions. This verified the correct size of the trispecific antibody as well as the success of the purification process (**Supplementary Figure 13B**).

Hydrophobic interaction chromatography (HIC) was performed comparing the bispecific and trispecific variant. While trispecific antibodies eluted at a later retention time, it showed a uniform peak indication no semi-paired species like half antibodies, Hole-Hole homodimers or missing light chains (**Supplementary Figure 14A**). SEC analysis revealed little aggregation and a high uniformity of the analytes (**Supplementary Figure 14B**). Comparing the retention times with a molecular weight standard underlined the correct size of the produced variants, which is in accordance to the SDS-PAGE analysis (**Supplementary Figure 14C**).

**Figure 4:**
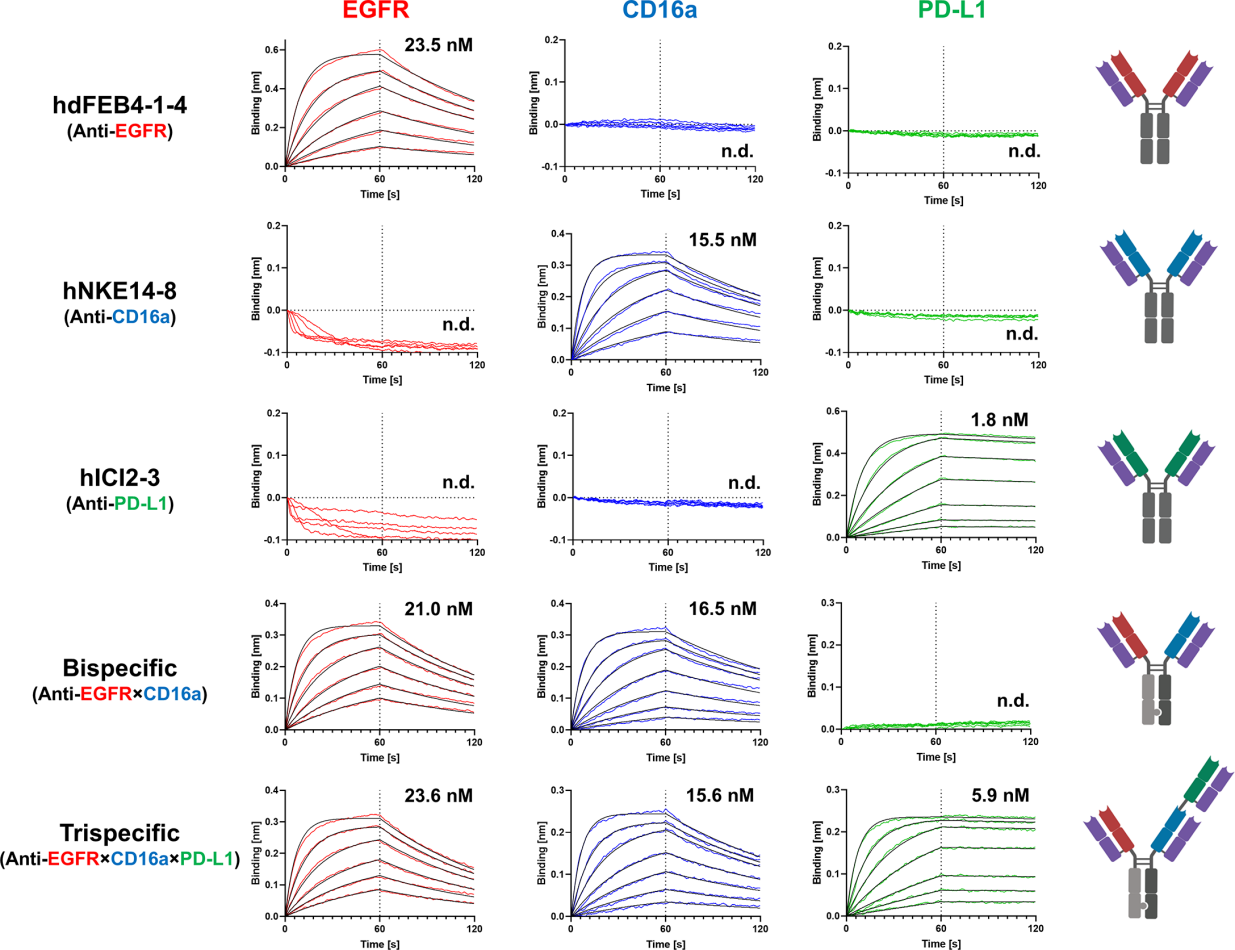
BLI-measurements of humanized mono-, bi-, and trispecific antibodies against EGFR, CD16a, and PD-L1. While all antibodies bind exclusively to their respective target with distinguished affinity, the trispecific antibody binds all three antigens.

BLI measurements were performed to determine the affinity of the respective antibodies to all three targets of interest. Variants hdFEB4-1-4, hNKE14-8, and hICI2-3 only bound their respective antigen and showed no binding to any other target of interest. The bispecific hdFEB4-1-4×hNKE14-8 bound EGFR and CD16a with an affinity comparable to its humanized parental variants and displayed no affinity towards PD-L1. Only the trispecific variant was able to bind all three antigens with high affinity (**Figure 4**).

**Figure 5:**
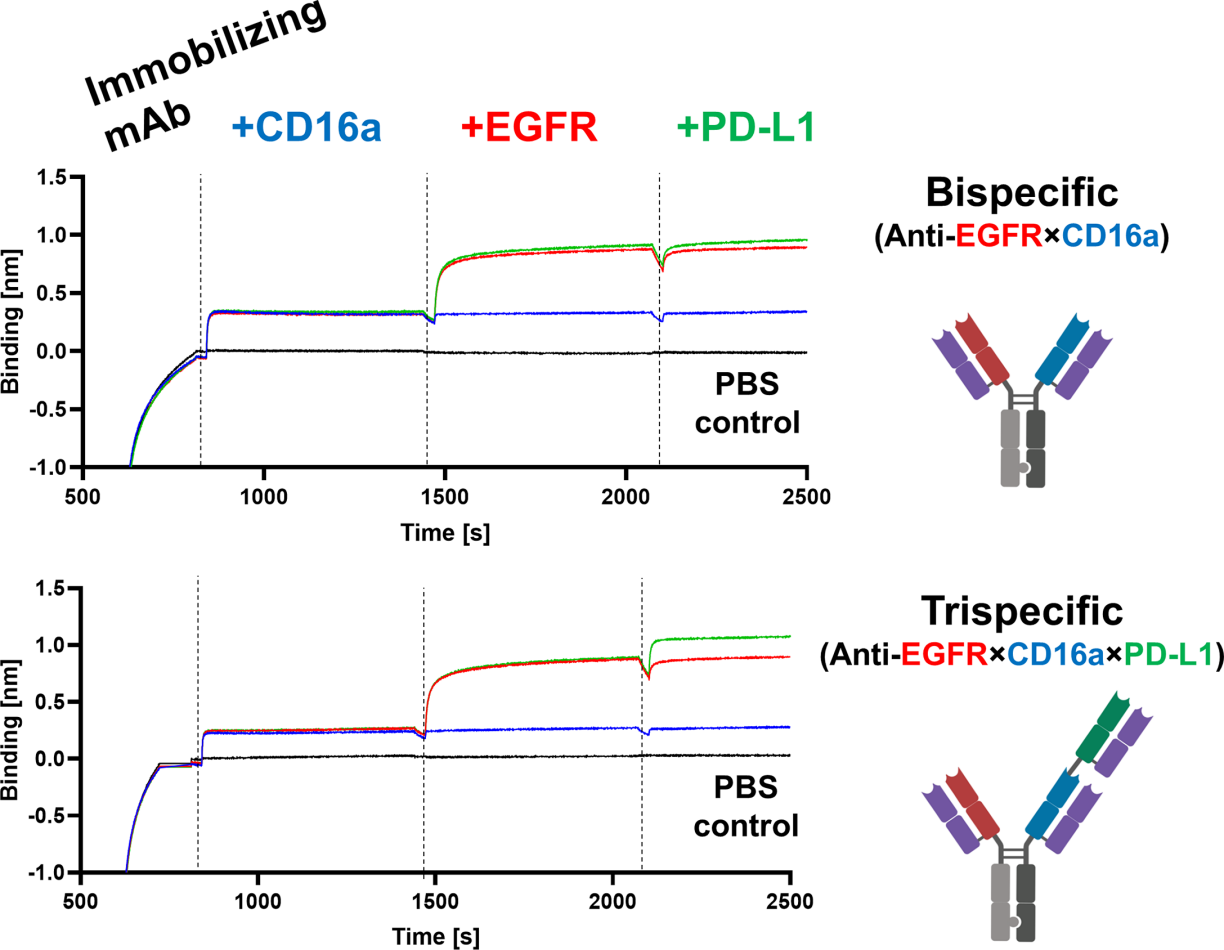
Characterization of bi- and trispecific humanized antibodies. BLI-assisted simultaneous binding assay. The bi- or trispecific antibodies were loaded onto biosensors, and antigens are added step-wise, revealing EGFR×CD16a bispecific or EGFR×CD16a×PD-L1 trispecific binding, respectively.

To mediate EGFR- and PD-L1-blockage, as well as effector cell recruitment, simultaneous binding of all target proteins is crucial. To test this mode of action, the antibodies of interest were loaded onto AHC biosensors and subsequently incubated in a consecutive manner with all three target proteins of interest, adding one antigen at a time. While hdFEB4-1-4, hNKE14-8, and hICI2-3 again showed only binding towards their respective target protein (**Supplementary Figure 15**), the bispecific variant showed simultaneous binding to EGFR and CD16a. Solely the trispecific variant was able to simultaneously bind all antigens at the same time, revealing true trispecific binding modalities (**Figure 5**).

As the trispecific antibody is intended to facilitate an elevated tumor specificity due to simultaneous binding to EGFR and PD-L1 compared to the bsAb solely targeting EGFR, cell-binding experiments were performed on EGFR/PD-L1 double-positive A431 cells using flow cytometry as described before (55) (**Supplementary Figure 16A**). Due to its bivalency, the trispecific constructs exhibited a stronger cell binding, in comparison to the bispecific variant (**Supplementary Figure 16B**).

### Cell-Based PD-L1 Blockage Reporter Assay

**Figure 6:**
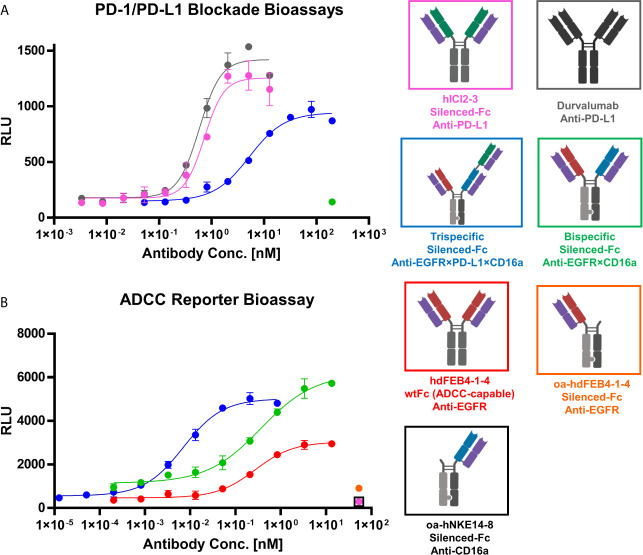
PD-1/PD-L1 blockage and ADCC cell-based reporter assays. **(A)** PD-1/PD-L1 blockage assay. hICI2-3 (pink), durvalumab (gray) were tested in comparison to the trispecific construct (blue) and the bispecific construct (green). EC_50_ values: durvalumab, 586 pM; hICI2-3, 728 pM; trispecific, 5.2 nM. **(B)** ADCC reporter bioassay. Bivalent hdFEB4-1-4 with a wildtype IgG1 Fc (red) and the bispecific construct with the LALA mutation were tested in comparison to the trispecific antibody. As controls, one-armed hdFEB4-1-4 (orange), one-armed hNKE14-8 (black), and hICI2-3 were tested. EC_50_ values: hdFEB4-1-4 (wtFc), 271 pM, bispecific antibody, 362 pM; trispecific antibody, 7.0 pM. A&B) Luciferase activity is plotted against the logarithmic antibody concentration. All measurements were performed in duplicates, and the experiments were repeated at least three times, yielding similar results.

To verify the PD-L1 blocking activity of the generated antibodies in a cell-based context, the Promega PD-L1 blockage assay kit was utilized. hICI2-3 showed efficient blockage of the PD-1:PD-L1 interaction comparable to durvalumab, underlining its prominent feature as a checkpoint inhibitor. The trispecific antibody showed significant PD-L1 blocking, even though to a less dominant effect compared to hICI2-3. This diminished EC_50_ value most probably originates in the monovalent binding of PD-L1 by the trispecific antibody, compared to the bivalent binding of the parental hICI2-3. The bispecific anti-EGFR×CD16a antibody, lacking the PD-L1 specific binding arm, does not interfere with the PD-1:PD-L1 interaction even at high concentrations (**Figure 6A**).

### Antibody-Dependent Cell-Mediated Cytotoxicity Reporter Assay

To investigate immune cell stimulation and efficiency of cell killing comparing the different antibody variants, the Promega ADCC luminescent reporter assay was used. As target cells, EGFR/PD-L1 double-positive A431 cells were utilized. The oahdFEB4-1-4 and oahNKE14-8 variants, as well as hICI2-3, all exhibiting the LALA mutation, as expected failed to mediate ADCC effects at high concentrations. The bispecific variant, simultaneously binding to EGFR on A431 target cells and CD16a on effector cells, mediated an ADCC effect that showed a significantly higher fold of induction than the bivalent hdFEB4-1-4 exhibiting a wild type IgG1 Fc. This indicated the effector cell engaging properties of the hNKE14-8 Fab fragment in a bispecific construct. The trispecific antibody, able to additionally bind PD-L1 simultaneously to EGFR and CD16, showed the most potent ADCC effect, presumably due to bivalent target cell binding (**Figure 6B**).

## Discussion

In this study, we established a generic straightforward method to generate humanized bi- and trispecific common light chain antibodies derived from chicken and describe the first trispecific antibody utilizing a single cLC. To this end, we applied a novel, straightforward method for the affinity maturation of cLC-based heavy-chain only binders by light chain shuffling. Furthermore, we illuminate the usage of yeast surface display-assisted epitope-binning based screening (54), to identify highly potent immune checkpoint inhibitors.

While the six CDRs comprise 40 to 50 residues, previous studies demonstrated that only 18-19 amino acids shape the paratope and are involved in antigen binding (63–67). Usually, these residues are distributed between the heavy and light chain CDRs, but since FEB4 VH tolerates combination with various unrelated VL domains (54), the crucial residues are located in the heavy chain CDRs. Light chain shuffling is a commonly used method to improve antibody affinity (68, 69). To simplify the screening procedure, we deliberately introduced affinity lowering mutations in the FEB4 CDRs to be able to screen for regain of high affinity upon combination with novel light chains. After reverting the VH CDRs to the wildtype sequence, an affinity matured variant was generated, where the VL contributes to antigen binding. Even though the change in affinity is only five-fold, this affinity maturation process could be a valuable addition to the armory of light chain shuffling-based affinity maturation approaches.

We recently described a YSD-assisted epitope binning-based screening approach to isolate anti-EGFR antibodies exhibiting non-overlapping epitopes to generate a biparatopic antibody (54). In this study, we modified that approach to isolate antibodies exhibiting an overlapping epitope with the therapeutic antibody durvalumab. Durvalumab is an FDA-approved antibody that binds an epitope of PD-L1 that efficiently disrupts PD-L1/PD1 interaction. Hence, we aimed at isolating common light chain binders from chickens recognizing a durvalumab-like epitope. Epitope binning experiments utilizing BLI proved that a panel of cLC antibodies was isolated and that these mAbs can effectively inhibit the interaction of PD-1 and PD-L1. This straightforward method could be applied to generate blocking antibodies against numerous immunoligands in an epitope-specific screening.

Our group previously showed that chicken-derived immune libraries, exhibiting common light chains can successfully be screened to isolate high affine binders targeting a large epitope space (53) and that these cLC mAbs can be utilized to construct biparatopic/bispecific antibodies exhibiting improved properties compared to their parental, monospecific counterparts (54). As those molecules were still of avian germline origin, we established an expeditious humanization technique, based on CDR-grafting and randomization of Vernier residues, to generate humanized antibodies with parental-like properties, high germline identity and low number of T cell epitopes (55). This strategy was used here for the first time to generate humanized bi- and trispecific molecules starting from avian antibodies.

**Figure 7:**
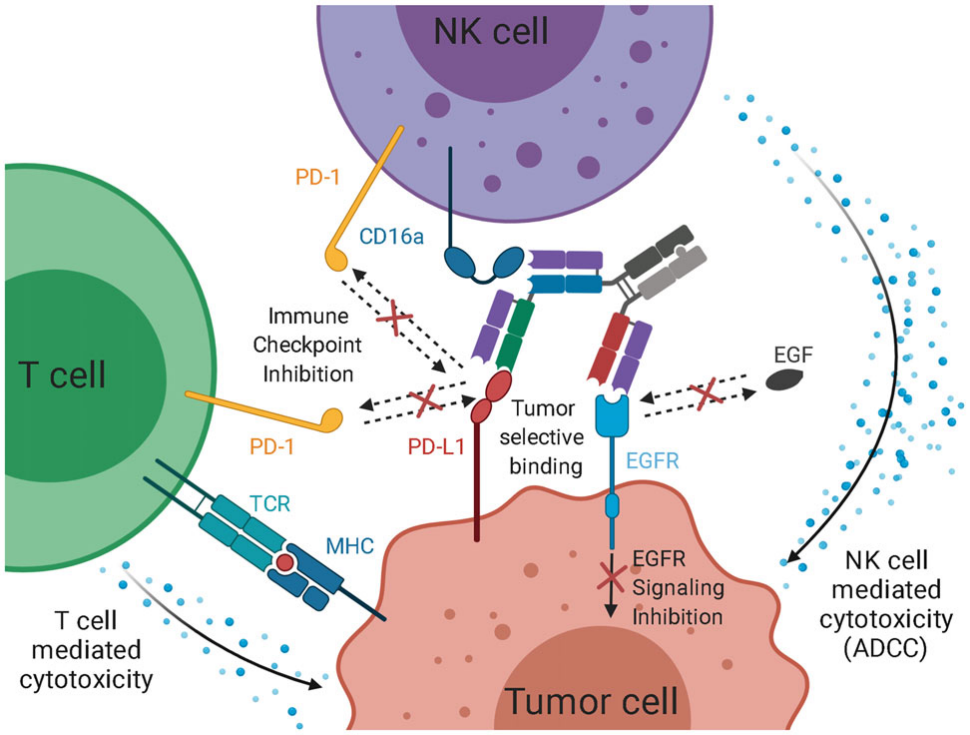
Schematic representation of the intended mode of action of the trispecific checkpoint inhibitor and natural killer cell engager. As EGF-binding is blocked, downstream signaling is inhibited. Simultaneously, PD-L1 is blocked, inhibiting interaction with PD-1 on T cells and NK cells. PD-L1 inhibition facilitates T cell-mediated cytotoxicity against the tumor. While the simultaneous binding of EGFR and PD-L1 mediates an elevated tumor specificity, the recruitment of cytotoxic NK cells *via* CD16a engagement, paired with checkpoint inhibition, leads to an effective ADCC reaction.

The 2 + 1 antibody format, often utilized in the context of TCBs, has been used for various bispecific antibodies and applications (45, 48, 50, 70–73). These TCBs share a common architecture, as the CD3-engaging Fab fragment is located directly on the Fc, N-terminally fused to an additional Fab arm. The other two Fab arms bivalently bind to the tumor cells. We adopted this general architecture and placed the CD16a-binding Fab arm directly on the Fc, N-terminally fused to the anti-PD-L1 Fab arm (**Figure 7**). The utilization of a CD16a-binding moiety, in contrast to a CD3 binding one could positively contribute to the safety profile of the molecule, as CD3 engagement is often associated with cytokine release syndromes and cytokine storms (74). However, there is a more profound clinical experience with CD3-specific antibodies. The CD3-specific antibody muromonab was the very first approved antibody in 1985, and with catumaxomab and blinatumomab, two CD3-specific bsAb were approved by the FDA (3, 75, 76). CD16a-specific antibodies, on the other hand, are in preclinical stages and only one CD16a-engager, AFM24, recently entered phase I/II studies (NCT04259450). Future clinical studies will reveal the true potential and safety profile of CD16a-engaging bi- and trispecific antibodies.

Notably, the inner anti-CD16a Fab arm of the trispecific construct exhibits binding kinetics similar to the parental monospecific antibody as well as to the bispecific variant. The ability to accept a N-terminal Fab-based fusion partner is most probably dependent on the shape of the paratope of the inner Fab. Prior studies utilizing tandem Fabs or 2 + 2 bispecifics found that for some mAbs their affinity is slightly decreased in this inner position, while for other antibodies no major impact on affinity was observed (77–82). This underlines the suitability of the hNKE14-8 Fab arm to be used in this 2 + 1 architecture. However, future studies need to test different trispecific formats and arrangements of cLC Fabs to elucidate the perfect domain orientation to facilitate the maximal efficacy.

In TCBs that made their way to the clinic, the correct light chain pairing is mediated by application of the CrossMab technology (45, 83, 84). Even though this positively contributes to the correct pairing, some antibodies may exhibit scrambled light chains and are challenging to purify from the correctly assembled pool. Moreover, antibody production requires expression of two heavy and two light chains and finding a production cell line with the desired expression level of all four strands to maximize yield and correct assembly can be a challenging task. In our approach, a single common light chain is utilized, circumventing additional engineering of the Fab and solely resulting in correctly paired light chains.

While bivalent binding has a positive effect on target cell engagement (70), it does not allow for a more defined selection of tumor cells. By utilizing two different Fab arms against EGFR and PD-L1, the trispecific molecule of this study might exhibit elevated tumor selectivity. Koopmans and coworkers demonstrated that an EGFR×PD-L1 bispecific antibody showed an elevated tumor specificity and tumor uptake (**Figure 7**) (41). Furthermore, the blockage of the PD-1:PD-L1 axis contributes to NK cell and T cell-mediated killing, as it is an immune checkpoint for both cell types (24–29).

However, in complex molecules like trispecific antibodies with intended synergistic effects, the affinity of the single Fab arms and their interplay might have a significant impact on safety and efficacy. To translate this molecule to further preclinical studies, the affinities of the anti-EGFR and anti-PD-L1 arms might need to be optimized to facilitate a maximal discrimination between single positive healthy cells and double positive malignant cells, while the blocking activity of both Fabs should not be compromised. Furthermore, the affinity of the anti-CD16a Fab might need to be adjusted to facilitate an optimal NK cell engagement with reasonable activation in respect to safety. While plenty of affinity tweaking technologies are described in literature (85–88), this is beyond the scope of this proof of concept study.

Furthermore, the utilization of an effector competent or a (glyco-) engineered Fc combined with the 2 + 1 architecture could mediate a stronger NK cell activation due to avidity in CD16a binding. However, this might also lead to crosslinking of an effector cell bound by the Fab to a second effector cell bound to the Fc, resulting in target cell independent activity, as known from bivalent CD3-targeting antibodies (89). To circumvent this risk, many NK cell engagers in the literature bind to CD16a in monovalent fashion (14, 17, 18). One the other hand, a competent or a (glyco-)engineered Fc in combination with a 1 + 1 bsAb can effectively engage NK cells (90). Still, NK cell activity is also dependent on the distance between the target and the effector cell (91–93). Therefore, depending on the epitope of a tumor-specific Fab, an adjacent anti-CD16a Fab arm engaging NK cells might mediate a more favorable target:effector cell distance compared to the an Fc. Furthermore, the generation of a stable glycoengineered cell line for the production of antibodies is laborious and analysis of antibody glycoforms remains complex (94–96). Nevertheless, this study presents a modular and straightforward method to generate human trispecific cLC antibodies, not limited to NK cell engagers, therefore paves the way for all kinds if 2 + 1 antibodies not restricted to certain antigens.

Geiger et al., recently demonstrated an elegant way to further elevate tumor specificity in the 2 + 1 format by fusing an anti-idiotypic scFv masking the CD3 binding Fab. Only after tumor-associated proteolytic cleavage of the linker, the CD3-specific Fab is unmasked and able to engage T cells, resulting in an elevated safety profile (97). A second possible engineering strategy to further elevate tumor specificity is to incorporate tumor-specific antigen binding. Mimoto and coworkers recently presented an antibody that bound hIL-6R only in the presence of ATP, which is found in high concentrations in the tumor microenvironment (TME), but only in low concentrations in healthy tissue (98). An additional method to elevate tumor specificity is to incorporate pH-responsive binding modalities into the antibody (99). While healthy tissue exhibits a nearly neutral pH around pH 7.4, the TME can exhibit a significantly reduced pH between pH 6.5 and pH 6.9 (100, 101). Our group previously demonstrated an efficient strategy to incorporate pH-responsive binding into a common light chain by histidine scanning and FACS-assisted screening of an YSD library. We confirmed in a human bispecific antibody, that only one of both Fab arms became pH-responsive. In contrast, the second Fab arm, paired with the identical his-doped cLC, remained pH-independently in its binding behavior (102). This technology could be applied to only facilitate CD16a-binding under acidic conditions as found in the TME, resulting in no NK cell engaging in healthy tissue and therefore contributing to tumor specificity.

## Material and Methods

### Plasmids and Yeast Strains

All plasmids, as well as their utilization for library generation by homologous recombination, Golden Gate cloning, or for expression, were previously discussed in detail (53–55). Yeast strains and their handling are described elsewhere (53, 102).

### Chicken Immunization

Chicken immunization and library generation were performed as previously described (56). In brief, two chickens (*Gallus gallus domesticus*) were immunized with CD16a or PD-L1, respectively. Five immunizations were performed for the CD16a-immunized animal on days 1, 14, 28, 42, and 56. For the first two immunizations, CD16a-Fc (produced in-house) was utilized, all subsequent immunizations were performed with a mixture of CD16a-Fc and TwinStrep-tagged CD16a (produced in-house). After the fourth immunization, the serum titer against both antigens was determined. The animal was sacrificed on day 63, followed by isolation of the spleen and RNA extraction. For the second chicken, an identical immunization plan was applied, utilizing PD-L1-Fc (PeproTech) as the antigen. All chicken immunizations, as well as sacrifice of the animal and subsequent RNA extraction from resurrected spleen cells, were performed at Davids Biotech GmbH. Experimental procedures and animal care were in accordance with EU animal welfare protection laws and regulations.

### Library Generation and Sorting Procedure

RNA, isolated from bursa fabricii and the spleen of immunized animals, was transcribed to cDNA utilizing SuperScript III Reverse Transcriptase (Invitrogen) following the manufacturer’s instructions as described previously (53, 56). Amplification of VH and VL domain genes, homologous recombination in yeast, yeast mating for library generation, as well as general yeast handling and induction of gene expression were performed as described elsewhere (53, 102).

For kinetic off-rate screening to identify affinity matured FEB4 variants by light chain shuffling and humanized dFEB4-1 variants, the respective libraries were stained with 1 nM EGFR-ECD-Fc for 10 min, washed three times, and then incubated with 1 µM EGFR-His for 30 min at RT. Only variants with a slow off-rate remained bound, and all other displayed Fabs are saturated with EGFR-ECD-His. Detection of Fc-tagged EGFR was done utilizing the anti-human Fc-PE secondary antibody (InVitrogen). An identical approach was conducted for the kinetic off-rate screen of humanized NKE14 variants, utilizing 1 nM CD16a-ECD-Fc and 1 µM TwinStrep-tagged CD16a.

### Reformatting, Expression, and Purification of Chimeric and Humanized Full-Length, One-Armed, Bispecific, and Trispecific Antibodies

Reformatting of isolated chicken-derived chimeric and humanized Fabs into standard IgG1 antibodies, one-armed variants, and bispecific variants were done by Golden Gate cloning as described previously (54). Reformatting into trispecific antibodies was done following the general architecture of cibisatamab (IMGT entry ID 10636). The hICI2-3 Fab (VH and CH1) was C-terminally fused with a partial hinge (EPKSCD), enabling the formation of a disulfide bond between the heavy and the light chain, followed by a (G_4_S)_2_-linker and the VH domain of hNKE14-8 (47). Cloning was done by amplification of both hICI2-3 Fab and the hNKE14-8 VH domain encoding sequences utilizing the primers depicted in **Supplementary Table 1**, followed by Golden Gate assembly utilizing an IgG1 LALA Hole His-tag Entry vector, similar as previously described (53, 54). All variants were expressed in Expi293F cells following the manufacturer’s protocol and subsequent purification was performed as described before (53, 54).

### Affinity Determination, Epitope Binning, Receptor-Ligand Competition and Simultaneous Binding Assay *via* Biolayer Interferometry

Affinity determination and epitope binning in the in-tandem setup were performed as previously described (54).

For the EGF competition assay, cetuximab, FEB4 or dFEB4-1 (10 µg/mL) were loaded onto AHC biosensors until a layer thickness of 0.7 nm to 1 nm was reached. Association was measured against 100 nM EGFR-ECD preincubated with 0 nM, 100 nM or 1000 nM EGF for 600 s.

For the Fc competition assay, NKE14 (10 µg/mL) was loaded onto Fab2G biosensors until a layer thickness of 0.7 nm to 1 nm was reached. Association was measured against 100 nM CD16a preincubated with 0 µM, 0.1 µM, 1 µM or 5µM scFv-Fc for 600 s.

For the PD-1 competition assay, durvalumab, ICI2, ICI12, and ICI13 (10 µg/mL) were loaded onto Fab2G biosensors until a layer thickness of 0.7 nm to 1 nm was reached. Association was measured against 250 nM PD-L1-ECD preincubated with 0 nM, 25 nM, 50 nM, 75 nM, 100 nM, 250 nM, 1000 nM amPD-1-Fc for 600 s.

For the simultaneous binding of multiple antigens to one antibody, the mAb of interest (10 µg/mL) was loaded onto AHC biosensors until a layer thickness of 1 nm was reached. Subsequently, the association against 1 µM CD16a-ECD was measured for 600 s. Next, 500 nM EGFR followed by 1 µM PD-L1-ECD were added, each over a course of 600 s. As controls, measurements with biosensors only incubated with CD16a-ECD and EGFR-ECD, only with CD16a-ECD or PBS, respectively, were performed.

All measurements were performed utilizing the Octet RED96 system (FortéBio, Molecular Devices) at 30 °C and 1000 rpm.

### Humanization

Humanization of chicken-derived antibodies was performed as described before (55). In brief, the CDR regions of the antibody of interest were grafted onto the human acceptor framework IGHV3-23 and IGHJ4 for the heavy and the IGLV3-25 and IGLJ2 for the light chain. Vernier residues, responsible for the correct orientation of the CDRs, namely residue 47, 49, 67, 75, 76, and 78 for the VH, and residue 46, 66, 69, and 71 for the VL were partly randomized by degenerated codons to either encode the human or the chicken amino acid at this position. The library was generated by fusion of oligonucleotides encoding the humanized VH and VL variants by PCR and subsequent subcloning into YSD vectors by Golden Gate as described before (103, 104). The Golden Gate reactions yielded bidirectional display plasmids encoding one humanized VH and VL domain. By transformation into EBY100 *S. cerevisiae* cells conducting electroporation, a library was generated, that could be screened for humanized variants exhibiting Vernier residues combination, allowing or the isolation of antibodies with parental biophysical properties (55).

### Nano DSF, Size Exclusion Chromatography and Hydrophobic Interaction Chromatography

Thermal stability by NanoDSF and Size Exclusion Chromatography was performed as described (53). For bi- and trispecific constructs, the SEC protocol was modified utilizing a 100 mM HEPES pH 6.8, 200 mM arginine buffer. For HIC, a 20 mM Tris pH 7.4, 1.5 M ammonium sulfate buffer A and a 20 mM Tris pH 7.4 buffer B were utilized with a gradient (0-100% buffer B) from 2.5 min to 37.5 min. The flow was 0.9 mL/min and a TSKgel Butyl-NPR column was used.

### Cell-Based PD-L1 Blockage Reporter Assay

For the cell-based checkpoint inhibitor assay, the Promega PD-1/PD-L1 Blockade Bioassays (J1250) was used, following the manufacturer’s instructions. A 2.5-fold dilution series of the antibodies of interest was applied. Testing concentrations were 3.35 pM – 12.8 nM for hICI2-3 and durvalumab, or 52.4 pM – 200 nM for the trispecific construct. The bispecific construct was used at a concentration of 200 nM. The assay was performed for six hours at 37°C and 5% CO_2_. Luciferase activity was plotted against the logarithmic antibody concentration. A variable slope four-parameter fit was utilized to fit the resulting curves. The assay was repeated three times, yielding comparable results.

### Antibody-Dependent Cell-Mediated Cytotoxicity Reporter Assay

A431 target cells were cultivated as described previously (54). The ADCC assay was performed utilizing the Promega ADCC Reporter Bioassay Kit (G7010) following the manufacturer’s instructions. The day before the assay, 12.500 A431 cells were seeded in a tissue culture-treated 96-well plate. A four-fold dilution series of the antibodies of interest was tested (0.2 nM – 13.3 nM for the bivalent hdFEB4-1-4 with a wildtype IgG1 Fc and the bispecific construct and 833 pM – 12.7 fM for the trispecific construct). One-armed hdFEB4-1-4, one-armed hNKE14-8, and hICI2-3 were tested at 53.3 nM. The assay was performed for six hours at 37°C and 5% CO_2_. Luciferase activity was plotted against the logarithmic antibody concentration. A variable slope four-parameter fit was utilized to fit the resulting curves. The assay was repeated three times, resulting in comparable results each time.

## Data Availability Statement

The antibody sequences presented in this article are not readily available because of upcoming patent applications. The sequences will become publicly available on https://tuprints.ulb.tu-darmstadt.de/ under the URN “urn:nbn:de:tuda-tuprints-178958” and on https://doi.org/10.26083/tuprints-00017895, respectively, at the latest in May 2023.

## Author Contributions

JB and HK and designed the experiments. JB, SC, and DF performed the experiments. JB, SC, DF, JG, and HK analyzed the data. DF, JG, and BH gave scientific advice. JB and HK wrote the manuscript. All authors contributed to the article and approved the submitted version.

## Funding

This work was supported by the Ferring Darmstadt Labs at Technical University of Darmstadt and by the department of GPRD at Ferring Holding S.A., Saint-Prex. The funders had no role in study design, data collection, and analysis, decision to publish, or preparation of the manuscript.

## Conflict of Interest

JG and BH were employed by the company Ferring Pharmaceuticals. JB, SC, and DF are employed by TU Darmstadt in frame of a collaboration project with Ferring Pharmaceuticals.

The remaining authors declare that the research was conducted in the absence of any commercial or financial relationships that could be construed as a potential conflict of interest.
