# Supplementary material for: Design of a Trispecific Checkpoint Inhibitor and Natural Killer Cell Engager Based on a 2 + 1 Common Light Chain Antibody Architecture
Source: Front Immunol. 2021 May 10;12:669496. doi: 10.3389/fimmu.2021.669496 (PMC8141644; doi:10.3389/fimmu.2021.669496)
Supplement: Supplementary file 1 [file DataSheet_1.pdf]

Supplementary Material

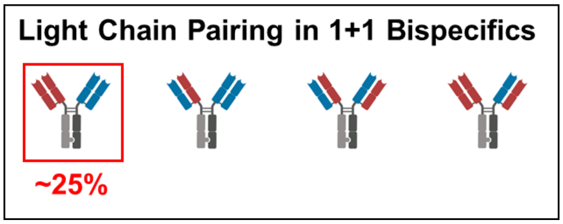

Supplementary Figure 1. Possible light chain pairing combinations in bispecific antibodies. In 1+1 bispecifics, only four different light chain pairing combinations are possible. The correctly paired variant is marked.

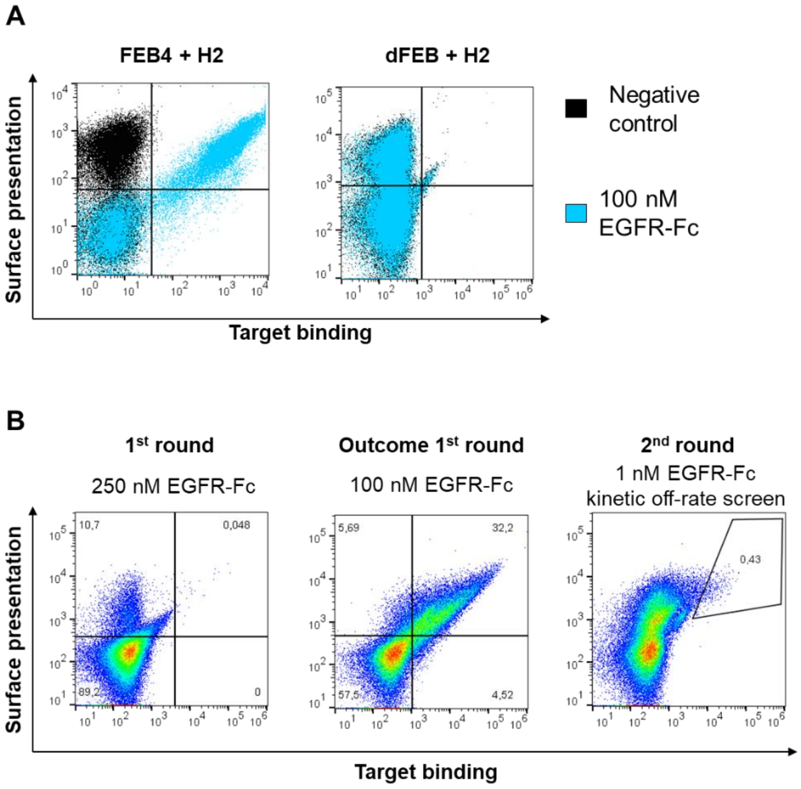

Supplementary Figure 2. Affinity maturation of FEB4. A) FACS plot of yeast cells displaying either FEB4 or the disrupted variant dFEB4 with its original H2 light chain. Negative controls without EGFR-Fc are black, while measurements including the antigen are shown in blue. B) Sorting of dFEB4 VH paired with the VL diversity for two rounds. A&B) Surface presentation is depicted on the y-axis utilizing the anti-human Lambda AF647, while EGFR-Fc binding is shown on the x-axis, using the anti-human Fc-PE secondary antibody.

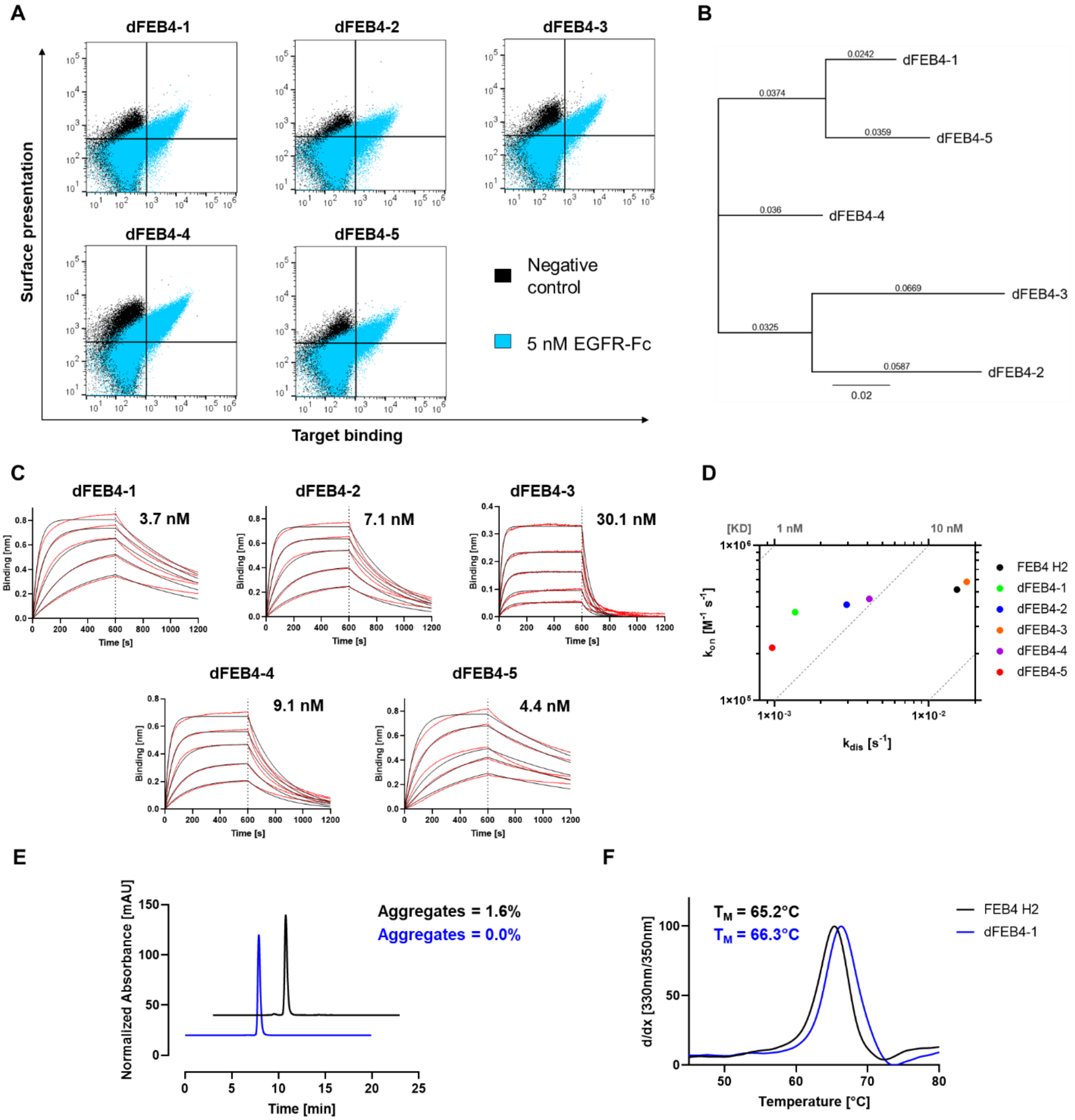

Supplementary Figure 3. Characterization of affinity matured FEB4 variants. A) Flow cytometric analysis of affinity matured antibodies enriched over two rounds of sorting. Negative controls without EGFR-Fc are depicted in black, while measurements including the antigen are shown in blue. Surface presentation is represented on the y-axis utilizing the anti-human lambda chain antibody AF647 labelled, while EGFR-Fc binding is shown on the x-axis, using the anti-human Fc-PE secondary antibody. B) Phylogenetic distance between isolated VL sequences. C) BLI measurements using the original FEB4 VH paired with the isolated VL sequences. D) Isoaffinity graph comparing the FEB4 VH paired with the original H2 light chain to FEB4 paired the isolated VLs from the shuffling approach. Comparison of SEC profiles (E) and NanoDSF measured melting temperatures (F) of FEB4 paired with either H2 (black) or dFEB4-1 (blue).

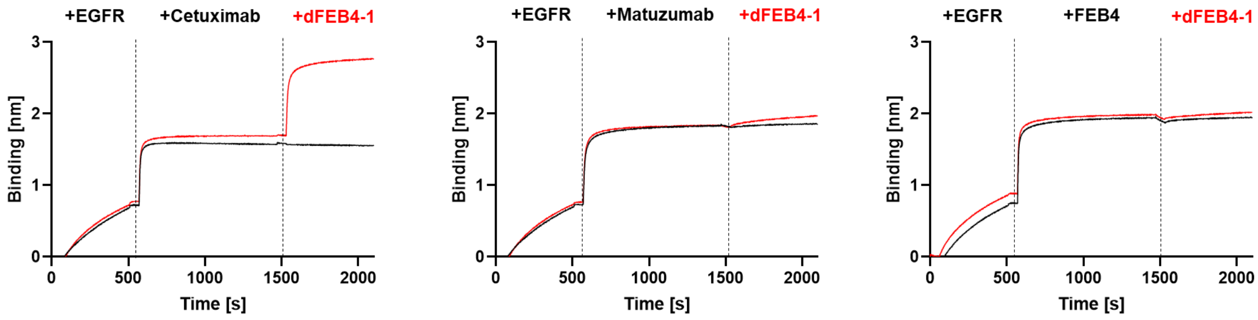

Supplementary Figure 4. Epitope binning of dFEB4-1. EGFR is immobilized utilizing His1K biosensors. Subsequently, either cetuximab, matuzumab, or FEB4 is applied, followed by incubation with either PBS or dFEB4-1.

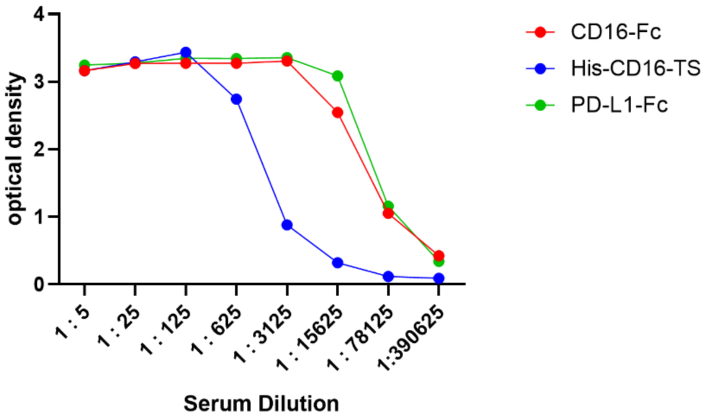

Supplementary Figure 5. Titer curves of immunized chickens. A serum sample was collected after the fourth immunization from two chickens, one immunized with CD16a-Fc and boosted with monomeric CD16a, and one immunized with PD-L1-Fc. A step-wise dilution of the serum was measured for antigen binding in an ELISA-based assay.

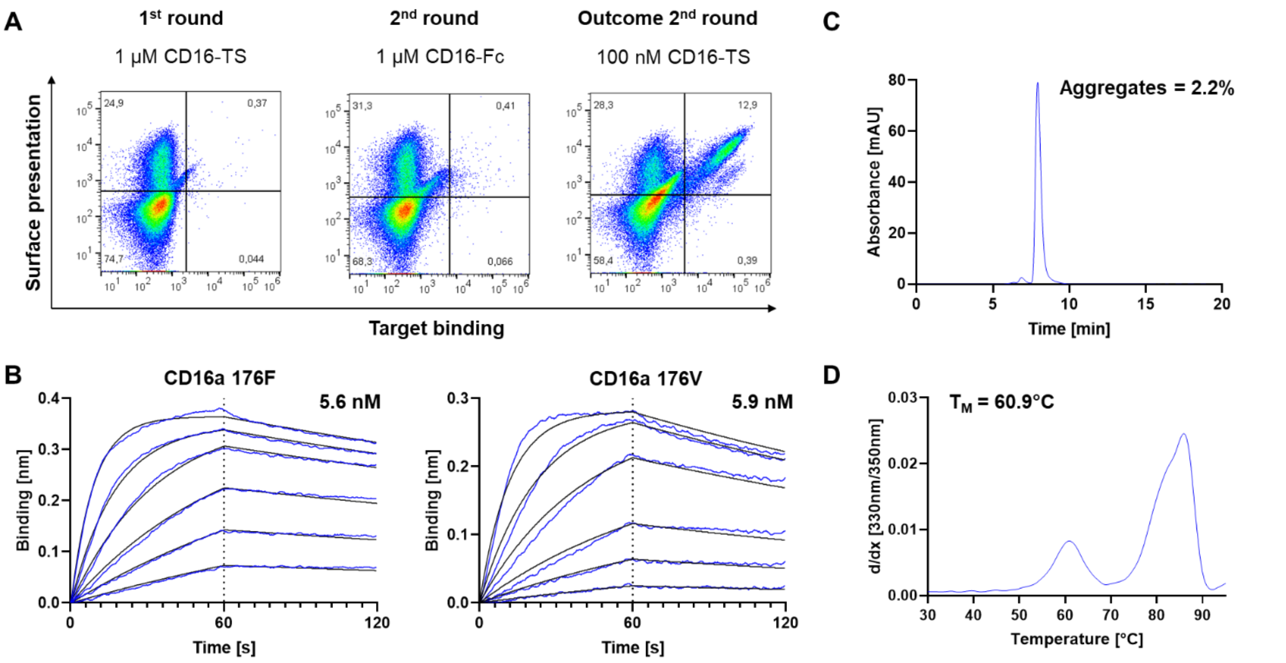

Supplementary Figure 6. Isolation and characterization of NKE14. A) Sorting of the CD16a yeast library. Surface presentation is depicted on the y-axis utilizing the anti-human lambda chain antibody AF647 labelled, while CD16a-Fc or CD16a-His binding is shown on the x-axis, using the anti-human Fc-PE or Streptavidin-APC secondary reagent, respectively. B) Binding kinetic of the resulting clone NKE14 to both CD16a isotypes. SEC profiles (C) and NanoDSF measured melting temperatures (D) of NKE14.

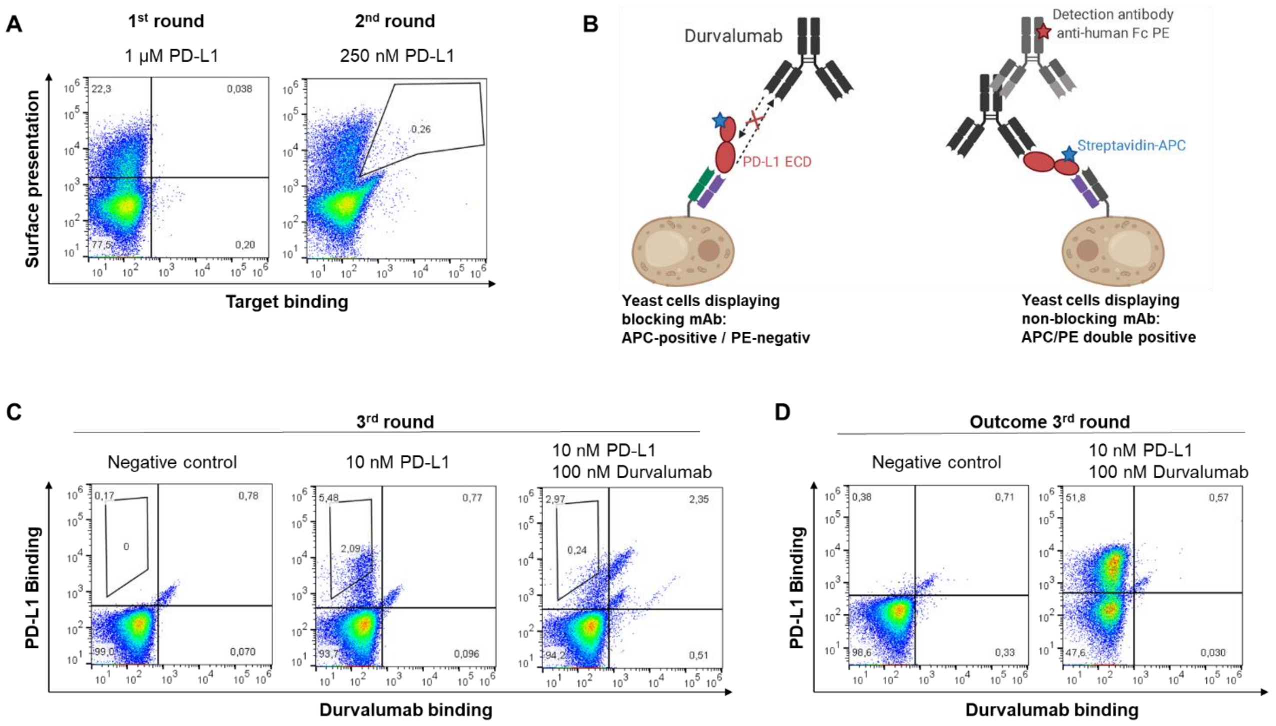

Supplementary Figure 7. Screening of PD-L1 library. A) Sorting of the PD-L1 yeast library. Surface presentation is depicted on the y-axis utilizing the anti-human Lambda PE, while PD-L1-His binding is shown on the x-axis, using the anti-His AF647 secondary antibody. B) Schematic representation of epitope binning based screening to isolate anti-PD-L1 antibodies with an overlapping epitope to durvalumab. C&D) Sorting of the PD-L1 yeast library. Target binding is depicted on the y-axis utilizing biotinylated PD-L1 ECD and Streptavidin-APC and durvalumab binding on the x-axis, using the anti-human Fc-PE secondary antibody.

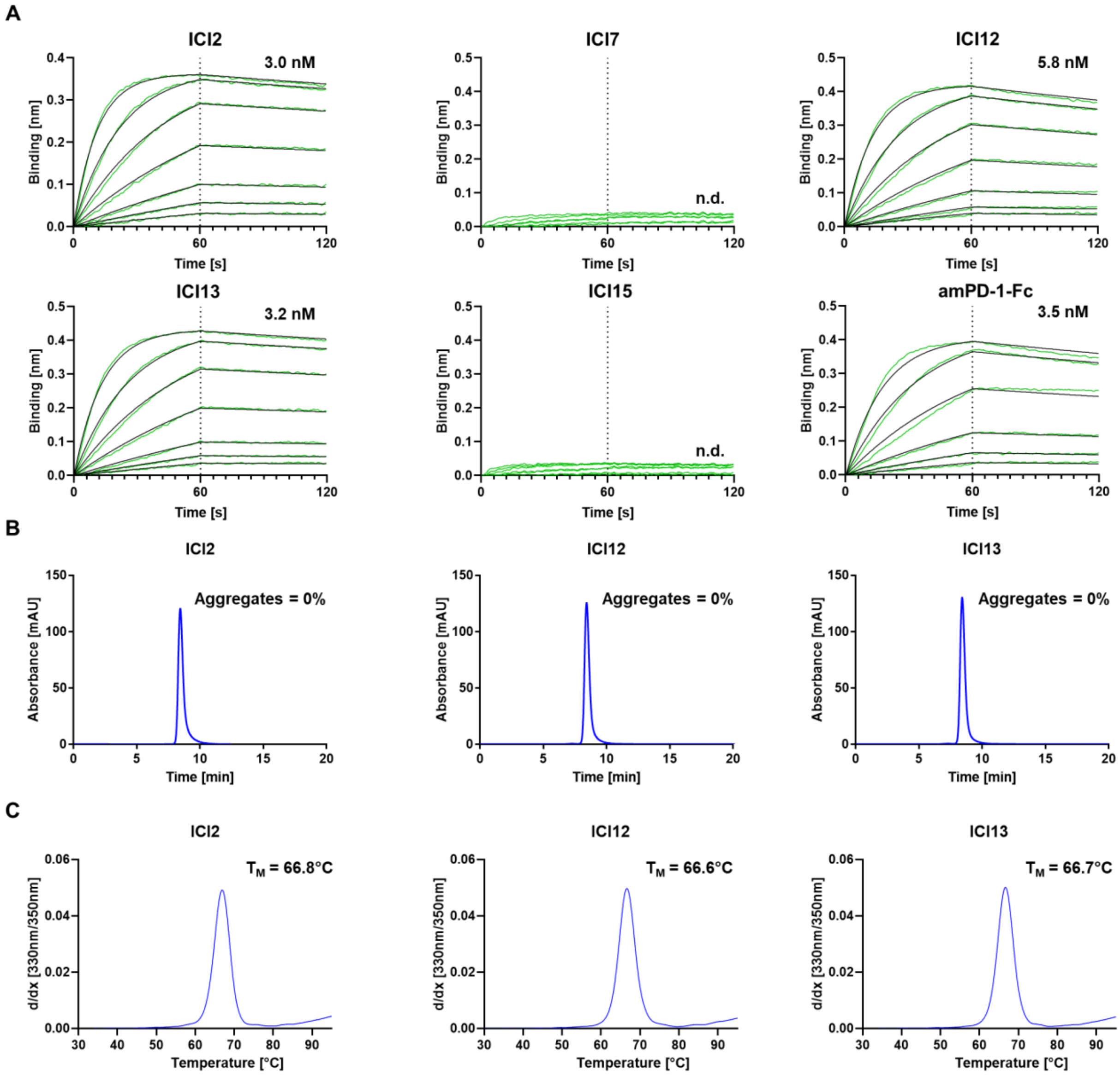

Supplementary Figure 8. Characterization of anti-PD-L1 ICI-variants. A) Binding kinetics of the resulting clones to PD-L1. SEC profiles (B) and NanoDSF measured melting temperatures (C) of ICI2, ICI12, and ICI13.

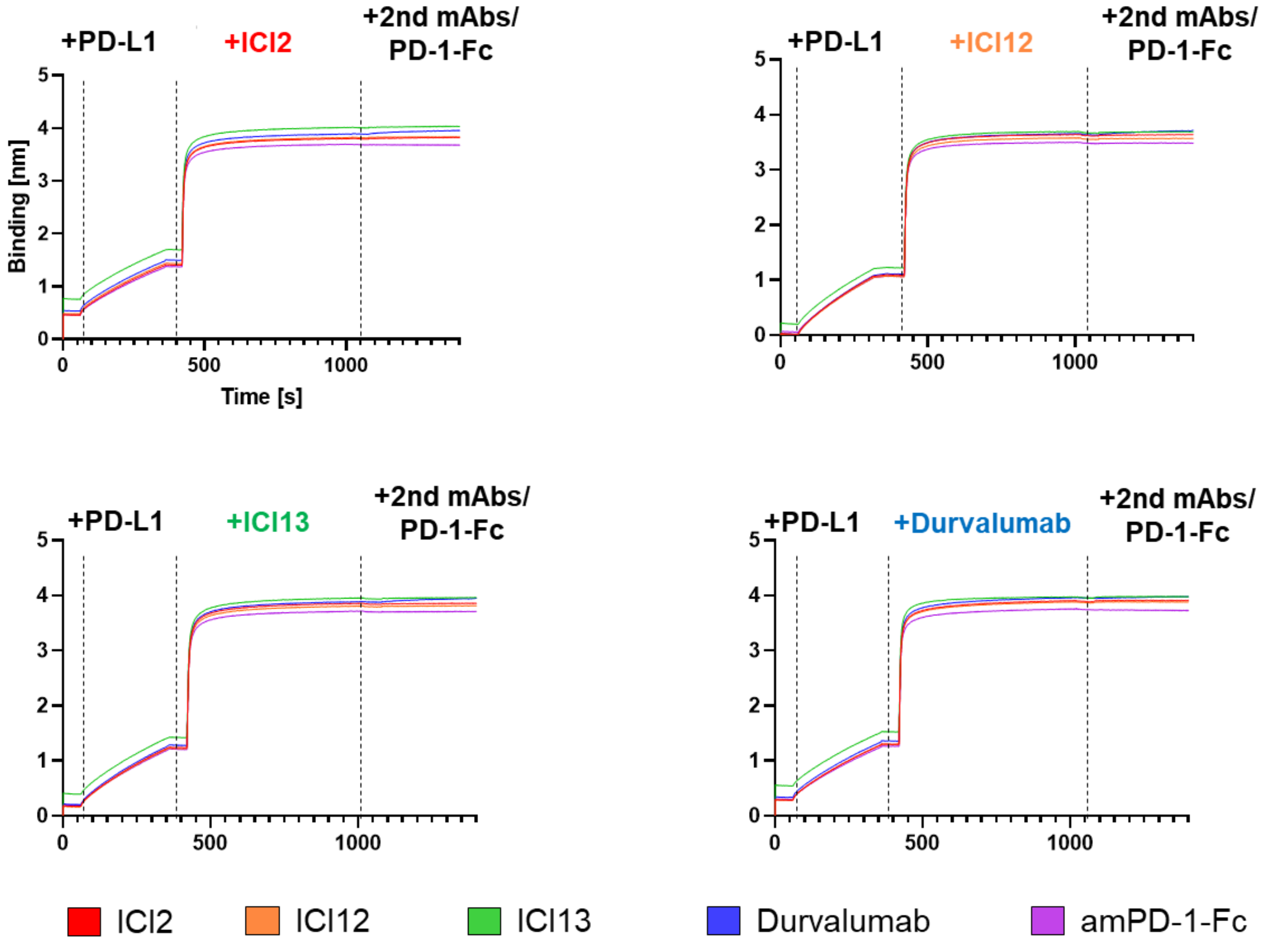

Supplementary Figure 9. Epitope binning of isolated ICI-variants. PD-L1-His is immobilized utilizing His1K biosensors. Subsequently, either ICI2, ICI12, ICI13, or durvalumab is applied, followed by incubation with either PBS or the secondary antibody. The applied secondary antibody is color-coded in the legend of the figure.

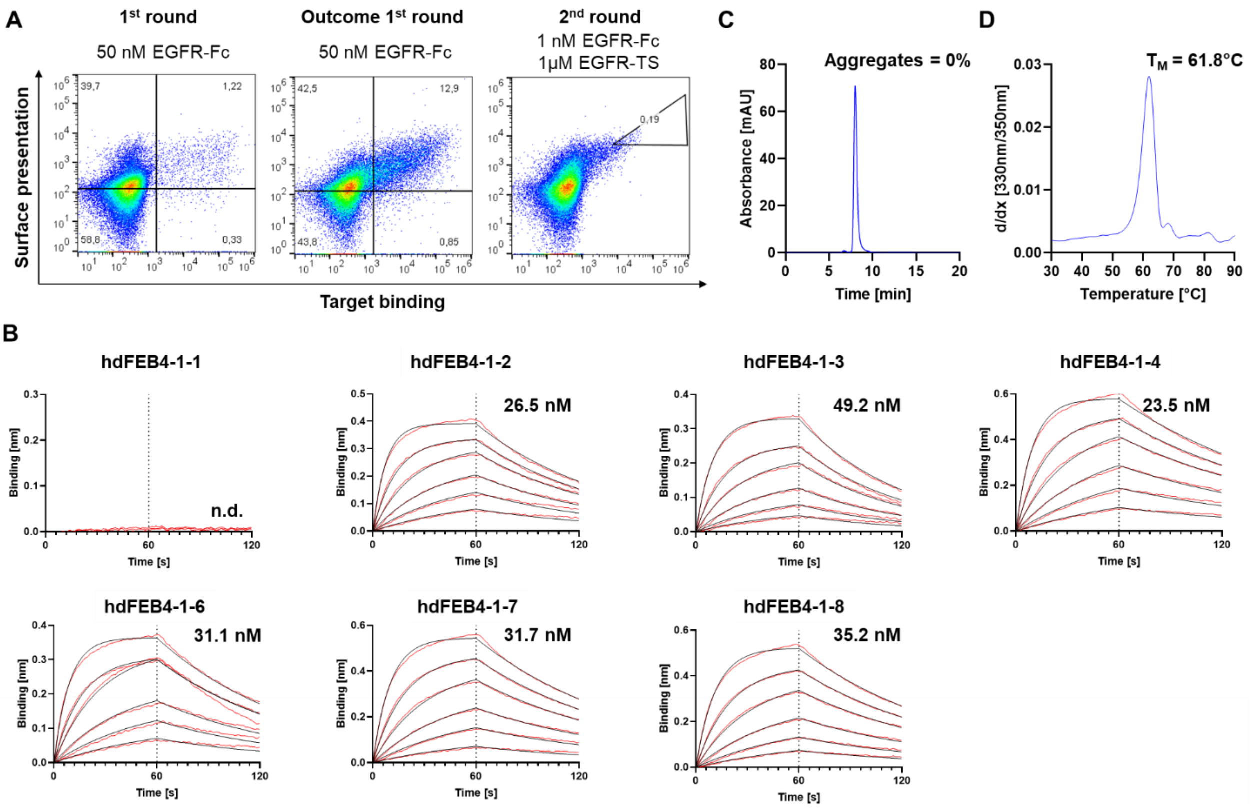

Supplementary Figure 10. Humanization of dFEB4-1. A) Sorting of humanized dFEB4-1 for two rounds. Surface presentation is depicted on the y-axis utilizing the anti-human Lambda AF647, while EGFR-Fc binding is shown on the x-axis, using the anti-human Fc-PE secondary antibody. B) Binding kinetics of the resulting clones to EGFR. SEC profiles (C) and NanoDSF measured melting temperatures (D) of hdFEB4-1-4.

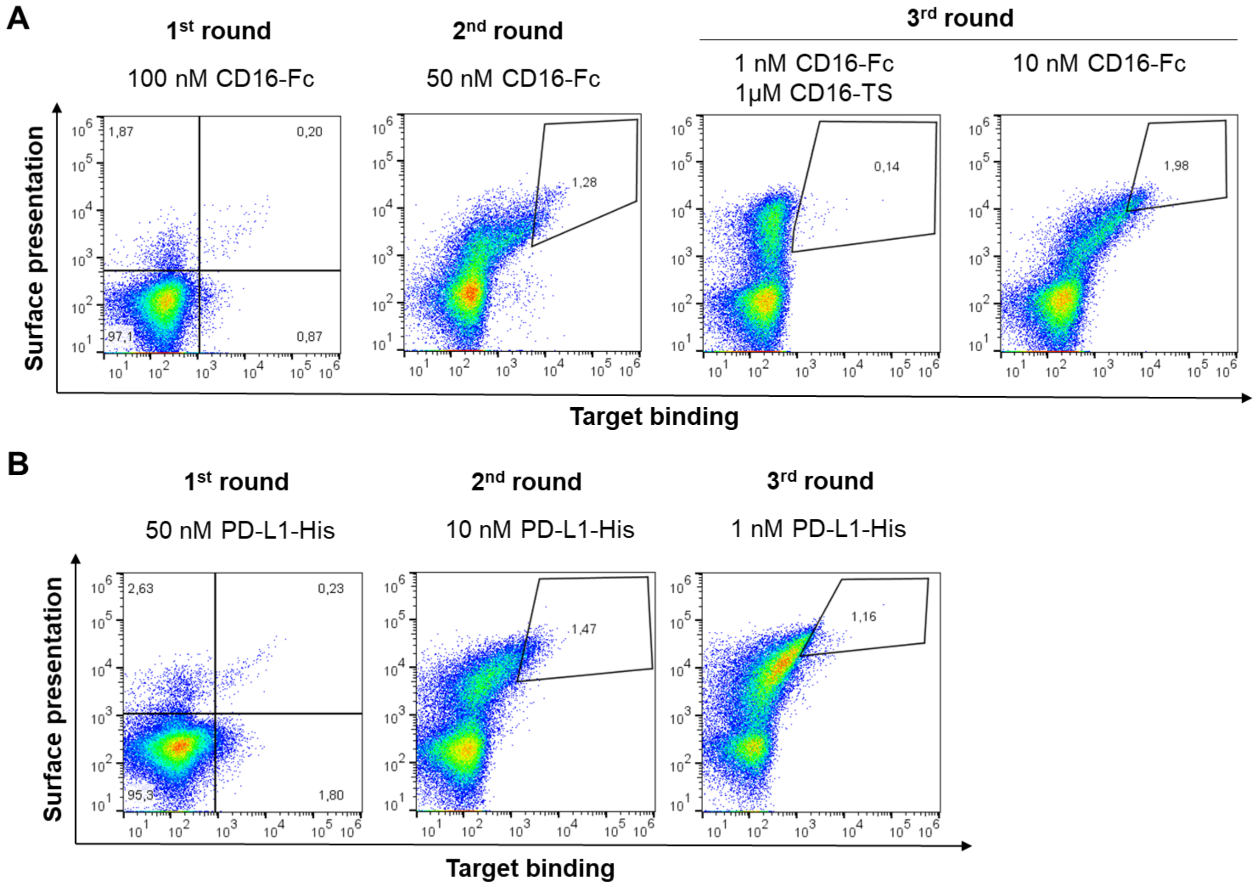

Supplementary Figure 11. Humanization of NKE14 and hICI2-3. Sorting of humanized NKE14 (A) or humanized ICI2 (B) variants for three rounds. For the NKE14 library, the third screening round was performed as a kinetic off-rate screen, as well as a standard screening round. Surface presentation is depicted on the y-axis utilizing the anti-human Lambda AF647. For NKE14, CD16a-Fc binding is shown on the x-axis, using the anti-human Fc-PE secondary antibody. For ICI2, PD-L1-His binding is shown on the x-axis, using the anti-His AF647 secondary antibody.

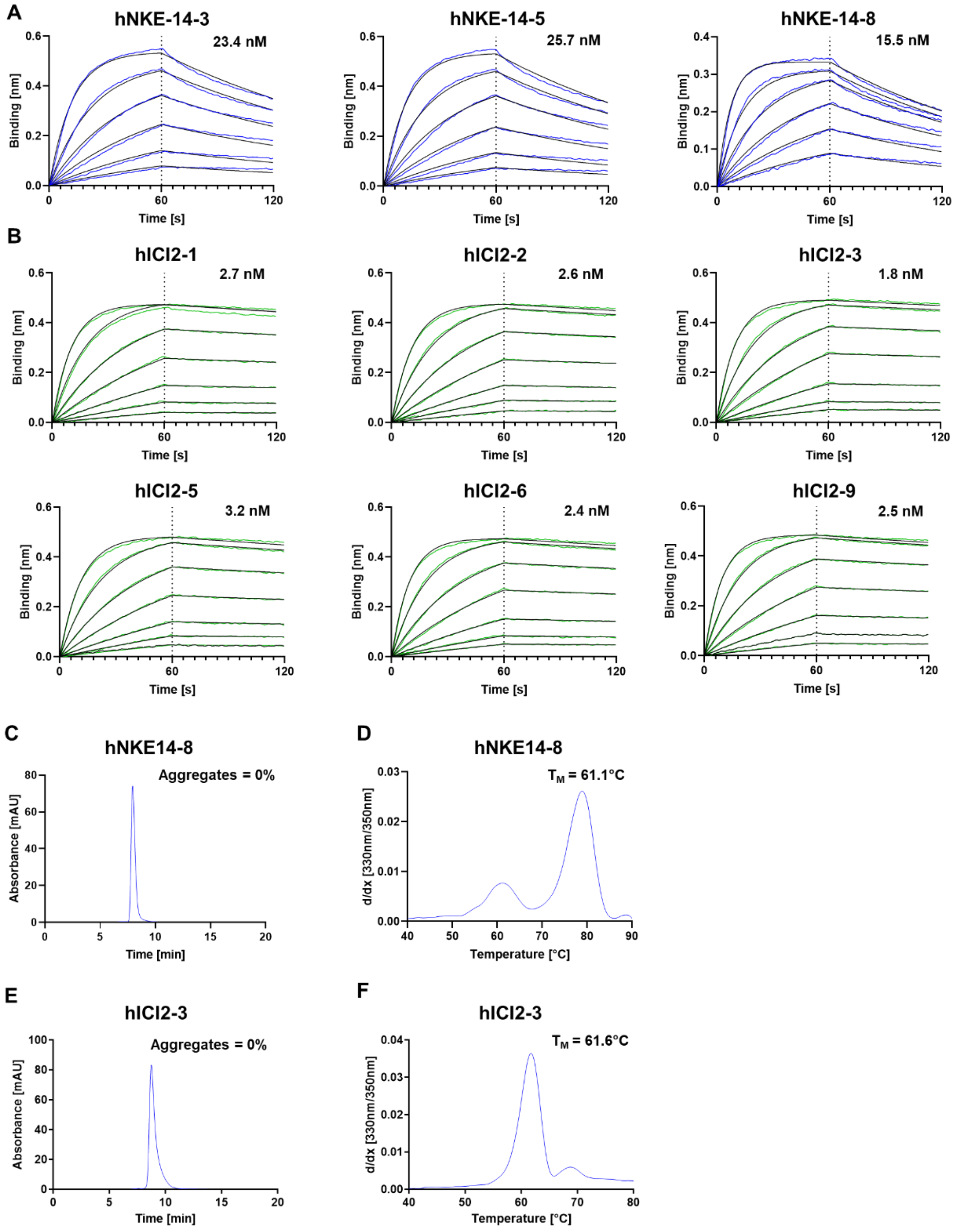

Supplementary Figure 12. Characterization of humanized NKE14 and ICI2 variants. Binding kinetics of the humanized NKE14 variants to CD16a (A) or humanized ICI2 variants to PD-L1 (B). SEC profiles and NanoDSF measured melting temperatures of hNKE14-8 (C, D) and hICI2-3 (E, F).

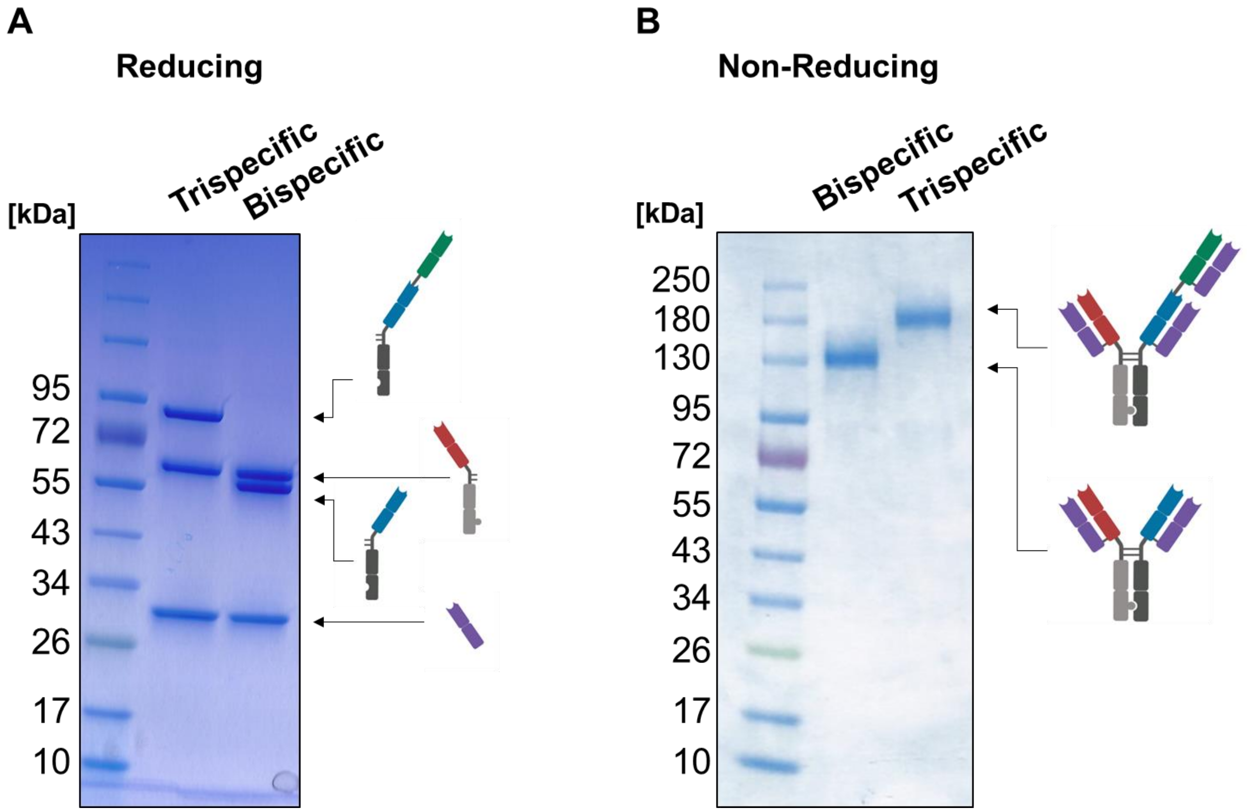

Supplementary Figure 13. SDS-PAGE analysis of the bi- and trispecific antibodies under reducing (A) and non-reducing (B) conditions revealed high purity and the expected molecular weights.

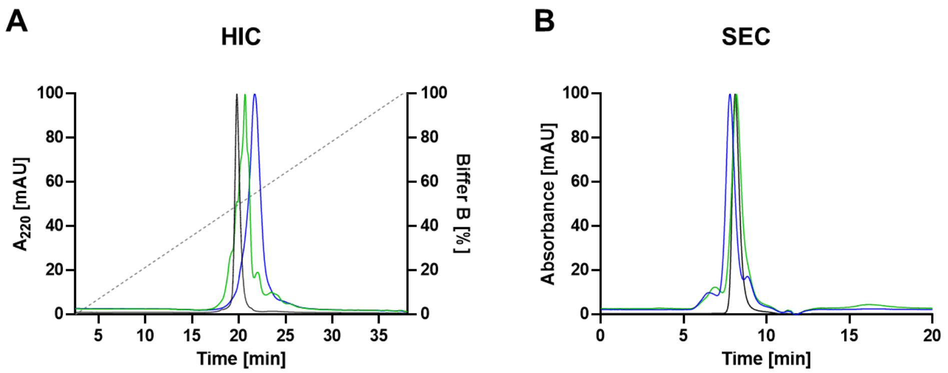

C

Antibody	Purity [%]	Theoretical mass [kDa]	SEC mass [kDa]
**Isotype**	100	146.0	149.3
**Bispecific**	95.4	148.1	140.1
**Trispecific**	86.7	195.2	200.9

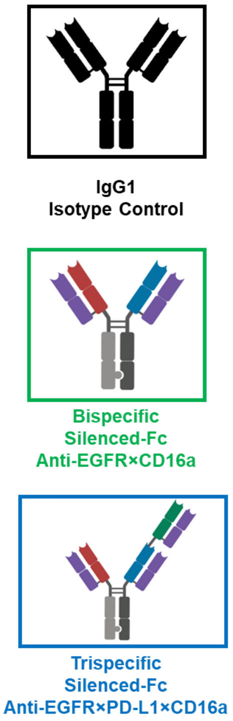

Supplementary Figure 14. Chromatographic characterization of bi- and trispecific variants. Comparison of an IgG1 isotype control with the bi- and trispecific variants utilizing hydrophobic interaction chromatography (A) and size exclusion chromatography (B). The colors of the profiles are according to the legend on the right side. C) The purity of the main peak of the SEC is depicted, along with the theoretical and the calculated mass of the analyte, based on a molecular weight standard.

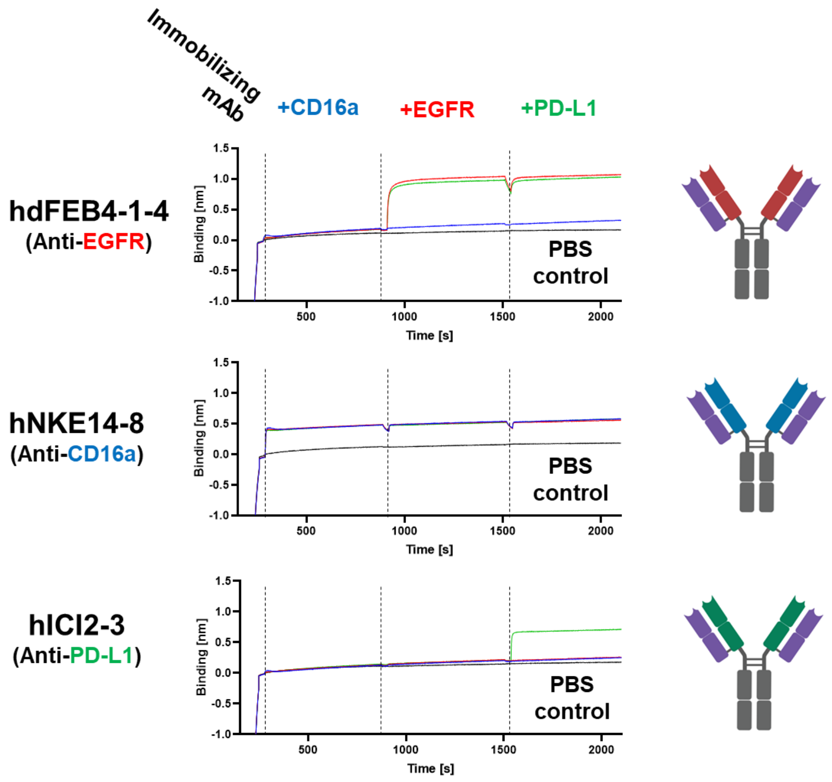

Supplementary Figure 15. Binding characterization of humanized monospecific antibodies. BLI-assisted simultaneous binding assay. The monospecific antibodies are loaded onto biosensors, and antigens are added step-wise, revealing monospecific binding to their respective antigen.

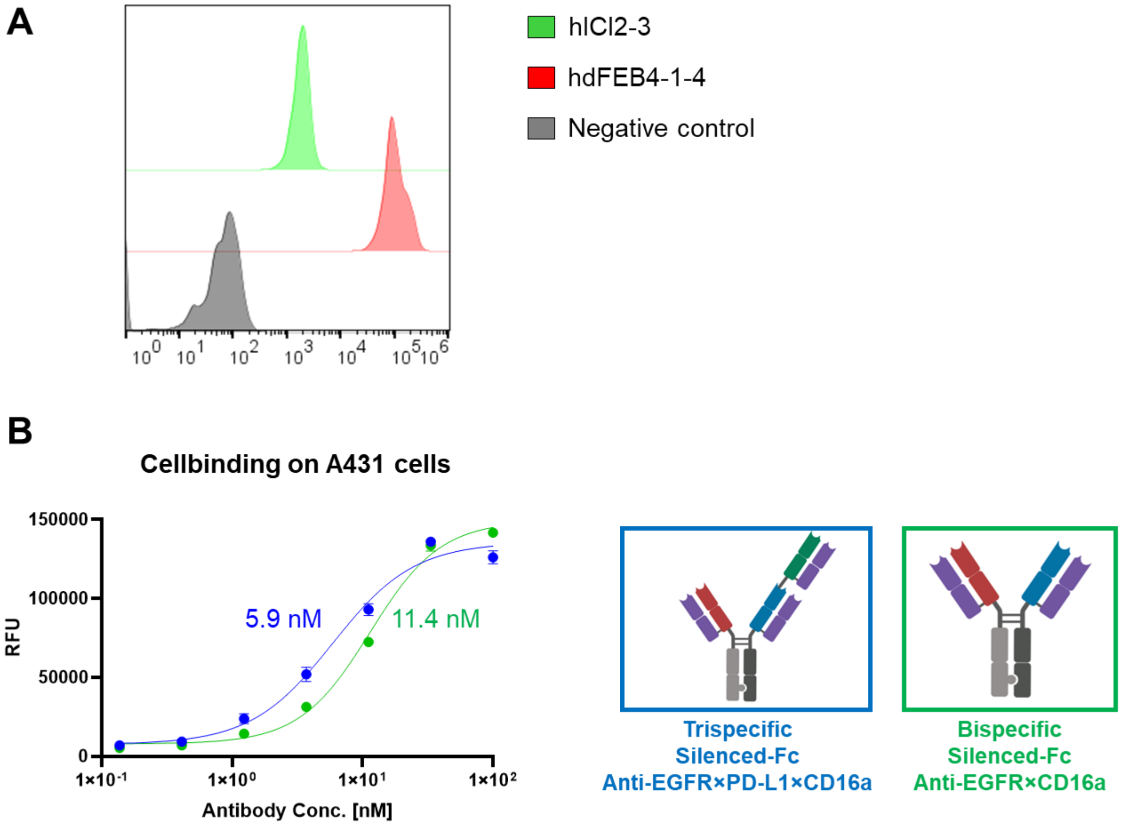

Supplementary Figure 16. EGFR/PD-L1 expression and cell binding. A) EGFR and PD-L1 expression on A431 cells. Cells were stained with either 100 nM hICI2-3 or hdFEB4-1-4 and binding was verified by using the anti-human Fc PE detection antibody. The negative control is shown in gray, while the anti-EGFR staining is shown in red and the anti-PD-L1 staining in green. B) Cell titration of the bispecific (green) and the trispecific (blue) construct on EGFR/PD-L1 double positive A431 cells. Antibody concentration ranged from 0.14 nM to 100 nM utilizing a threefold dilution. A variable slope four-parameter fit was utilized to fit the resulting curves. The assay was repeated twice, yielding comparable results. The EC_{50} values are indicated.

Supplementary Table 1. Primer sequencing for reformatting of hICI2-3 and hNKE14-8 into a “head-to-tail” fusion. Golden Gate overhangs encoding SapI restriction sites are highlighted in blue.

**Primer name**	**Template**	**Sequence 5’→3’**
HumRe VH Fr1 for	hICI2-3	AAAAAGCTCTTCA AGTGAGGTGCAGCTGTTGGAGTCTGGGGG
2+1 outer Fab CH1 rev	hICI2-3	TTTTTTGCTCTT CCTGAGCCGCCTCCCCCGTCACAAGATTTG GGCTCAACTCTC
2+1 inner hFab for	hNKE14-8	AAAAAGCTCTTC CTCAGGCGGCGGCGGGTCGGAGGTGCAGC TGTTGGAGTCTGGGGG
HumRe VH Fr4 Fab rev	hNKE14-8	TTTTTTGCTCTTCT GGCTGAGGAGACGGTGACCAGGGTTCC
